# EDIT-B consortium: an external pan-European validation of an innovative machine learning-based test for bipolar disorder diagnosis

**DOI:** 10.1136/bmjment-2026-302859

**Published:** 2026-10-05

**Authors:** Dinah Weissmann, Nicolas Salvetat, Christopher Cayzac, Francisco Checa-Robles, Diana Vetter, Francisco Santos Schneider, Lara Walczer-Baldinazzo, Giovanna Ruggeri, Maurizio Ferrari, Andrea Miranda-Mendizabal, Victor Chavarria, Oscar Soto-Angona, Juan Zambrano, Jeff Zarp, Anna Gimenez-Palomo, Marc Valenti, Josep Maria Haro, Lars Vedel Kessing, Chantal Henry, Eduard Vieta

**Affiliations:** 1 Alcediag/Sys2Diag UMR9005 (CNRS/Alcen), Montpellier, France; 2 Genetic Unit, SYNLAB Italia Srl, Castenedolo, Italy; 3 IRCCS, SYNLAB SDN, Naples, Italy; 4 Institut de Recerca Sant Joan de Déu, Esplugues de Llobregat, Spain; 5 Mental Health Networking Biomedical Research Centre (CIBERSAM), Madrid, Spain; 6 Parc Sanitari Sant Joan de Déu, Sant Boi de Llobregat, Spain; 7 Research Network on Chronicity, Primary Care, and Health Promotion and Prevention (RICAPPS), Esplugues de Llobregat, Spain; 8 Université Paris Cité, Paris, France; 9 GHU Paris Psychiatrie & Neurosciences, Paris, France; 10 Copenhagen Affective Disorder Research Center, Copenhagen, Denmark; 11 Copenhagen Psychiatric Center, Hellerup, Denmark; 12 Bipolar and Depressive Disorders Unit (IDIBAPS), Hospital Clinic de Barcelona, Barcelona, Spain; 13 Department of Clinical Medicine, University of Copenhagen, Copenhagen, Denmark; 14 Institute of Neurosciences of the University of Barcelona (UBNeuro), Barcelona, Spain

**Keywords:** Psychiatry, Machine Learning, Bipolar and Related Disorders, Depressive Disorder, Mood Disorders

## Abstract

**Background:**

Differentiating bipolar disorder (BD) from major depressive disorder (MDD) during major depressive episodes remains a significant challenge. EDIT-B is an in vitro diagnostic test based on machine learning (ML) method integrating clinical metadata with adenosine-to-inosine (A-to-I) RNA editing signatures in eight genes (*GAB2*, *IFNAR1*, *IL17RA*, *LYN*, *MDM2*, *PRKCB*, *PTPRC*, *ZNF267*) to differentiate BD from MDD.

**Objective:**

The objective of this study is to assess and confirm the diagnostic performance of EDIT-B in a new, independent, double-blind, external, multicentre European cohort.

**Methods:**

We evaluated EDIT-B results across four European centres (Spain, France, Denmark) compared with physician diagnoses. Explainability and sensitivity analyses were performed to identify the primary drivers of the model.

**Findings:**

In 393 patients with current major depressive episode (238 MDD; 155 BD), EDIT-B demonstrated robust performance: area under the curve-receiver operating characteristic of 0.873 (95% CI 0.837 to 0.909), sensitivity of 82.6% (95% CI 75.7% to 88.2%) and specificity of 80.3% (95% CI 74.6% to 85.1%). Performances were consistent across sites and all stratified patient subgroups, corroborating previous results. Explainability analysis pointed to RNA editing biomarkers as the most important variables for EDIT-B diagnosis.

**Conclusion:**

This study confirms the diagnostic performances of EDIT-B showing that A-to-I RNA editing coupled with ML methods is a reliable approach to distinguish BD from MDD.

**Clinical implications:**

EDIT-B represents an innovative, reliable and complementary diagnostic tool supporting psychiatric practice and enhancing clinical outcomes with potential impact in reducing diagnostic delays, tailoring the therapeutic strategy and improving therapeutic alliances. These findings may represent a significant advance towards precision psychiatry, with potential to reduce misdiagnosis and inappropriate treatment.

**Trial registration number:**

NCT05603819.

**WHAT IS ALREADY KNOWN ON THIS TOPIC**

- Bipolar disorder (BD) is frequently misdiagnosed as major depressive disorder due to overlapping depressive symptoms, leading to diagnostic delays of up to a decade and important clinical consequences. Current diagnostic approaches rely on clinical assessment and lack specificity while few objective/biologically based tools are available for accurate early differential diagnosis.

**WHAT THIS STUDY ADDS**

- This study corroborates EDIT-B test performances for diagnosing BD during depressive episodes in a large, independent European multicentre replication cohort. This study also highlights the importance of RNA editing biomarkers for EDIT-B diagnosis. Sensitivity analyses show robust performances and no major difference among subgroups in all tested conditions including comparison between untreated and treated patients for several classes of psychiatric medications.

**HOW THIS STUDY MIGHT AFFECT RESEARCH, PRACTICE OR POLICY**

- EDIT-B addresses the need for a reliable and objective diagnosis of BD during depressive episodes. The EDIT-B test represents a significant advance in precision psychiatry, with potential to minimise misdiagnosis and diagnostic delays and to improve the accuracy and tailoring of treatment strategies.

## Introduction

 The differential diagnosis of bipolar disorder (BD) and major depressive disorder (MDD) remains a critical unmet need. Although both conditions are characterised by depressive episodes, their pathophysiological and treatment trajectories differ substantially. BD often initially presents with depression, leading to frequent misdiagnosis as MDD and an average diagnostic delay of nearly a decade.^1 2^ This delay has important clinical consequences, including inappropriate pharmacotherapy, increased risk of treatment-emergent mania and suicide.^3–5^ Traditional diagnostic approaches rely on symptomatology and structured interviews, lacking the precision to disentangle overlapping phenotypes.^6^ While certain clinical features (eg, early age at onset, mood lability and family history) may suggest BD, their predictive value remains limited. The limited availability of objective, biologically grounded tools for differential diagnosis impedes early and accurate identification of BD.

Recent advances in molecular psychiatry have focused on immune and oxidative pathways.^7^ Among emerging candidates, RNA editing, a dynamic mechanism modifying nucleotide sequences post-transcription, has shown promise. Dysregulated editing patterns were reported in BD and other neuropsychiatric disorders, implicating this process in mood regulation and neuroinflammation.^8 9^

In a previous study, EDIT-B, a blood diagnostic test, showed high sensitivity and specificity for distinguishing BD from MDD during major depressive episodes (MDE).^9^ EDIT-B uses a locked machine learning (ML) algorithm based on 22 RNA editing biomarkers from eight genes (*GAB2*, *IFNAR1*, *IL17RA*, *LYN*, *MDM2*, *PRKCB*, *PTPRC*, *ZNF267*) and clinical data for performing the diagnostic. The editing biomarkers were selected through a multistep strategy combining adenosine-to-inosine (A-to-I) RNA editome discovery, stringent filtering, biological/functional network analyses, and analytical and independent clinical validation.^9 10^ The biomarkers present interconnected pathways involved in neuroinflammation, microglial activation, immune signalling, oxidative stress and mitochondrial dysfunction, all implicated in the pathophysiology of BD (online supplemental figure 1).^2^ EDIT-B test is currently Conformité Européenne (CE) marked under the In Vitro Diagnostic Directive and already commercialised in some European countries.

Here, we present results and confirm the performance of EDIT-B in a novel external large-scale, independent, double-blind, pan-European cohort. The EDIT-B project is funded by the European Union (Grant No 220628/230125).

## Methods

### Study design and setting

The EDIT-B consortium study (ClinicalTrials.gov NCT05603819) is a double-blind, multicentre, cross-sectional study enrolling adults between June 2022 and September 2024 across four clinical sites: two in Barcelona (Spain), one in Paris (France) and one in Copenhagen (Denmark) (online supplemental file 2). Reporting followed the Standard Protocol Items: Recommendations for Interventional Trials (SPIRIT) guideline (online supplemental table 1). This study adhered to the AI-specific extension of Standards for Reporting of Diagnostic Accuracy Studies (STARD-AI) guidelines for reporting of AI-centred diagnostic test accuracy studies. Written informed consent was obtained from participants. This study was conducted in accordance with the Declaration of Helsinki (2013). An external central laboratory (SYNLAB Italia–Genetic Unit, Castenedolo, Italy) processed the blood samples and performed EDIT-B test on a dedicated secure web platform. SYNLAB was blinded from the clinicians’ diagnoses, and clinicians were blinded from EDIT-B results. The protocol of this study has been published in Miranda *et al*.^11^

### Clinical assessment

A total of 426 participants were screened for eligibility: 259 with MDD, 165 with BD and 2 with missing diagnostic. Eight participants did not meet the inclusion/exclusion criteria. Additionally, 14 participants were excluded for protocol deviations and 14 further participants were excluded for intended use or technical quality criteria. The final analytical set included 393 participants: 238 with MDD and 155 with BD (online supplemental figure 2). Eligibility criteria and sample size calculation are described in the online supplemental methods.

### EDIT-B in vitro diagnostic test

The panel of pre-mRNA sequences used by EDIT-B includes *GAB2*, *IFNAR1*, *IL17RA*, *LYN*, *MDM2, PRKCB*, *PTPRC* and *ZNF267*. EDIT-B genes were initially identified through an unbiased, data-driven analysis of the blood RNA editome.^10^

Details on the assay protocol and on the algorithm were previously described^9^ and are strictly the same used in this study. Briefly, blood samples were collected at the clinical centres using PAXgene blood RNA tubes and stored upright at room temperature (18−25°C) for 2 hours before freezing (−20°C). Patients were allowed to eat and to take their routine medicines before blood sampling. Fasting status and the time elapsed since the last medication administration were not documented. The samples were sent on dry ice every 2 months to SYNLAB using time- and temperature-controlled transport. Blood samples were treated in batch of randomly distributed patients. RNA was extracted and qualified before PCR, then the PCR product amplification was purified and quantified before indexing and pooling into a final library. The library was denatured, loaded and sequenced at standard concentrations on an Illumina NextSeq 500/550 sequencer (figure 1A).

**Figure 1:**
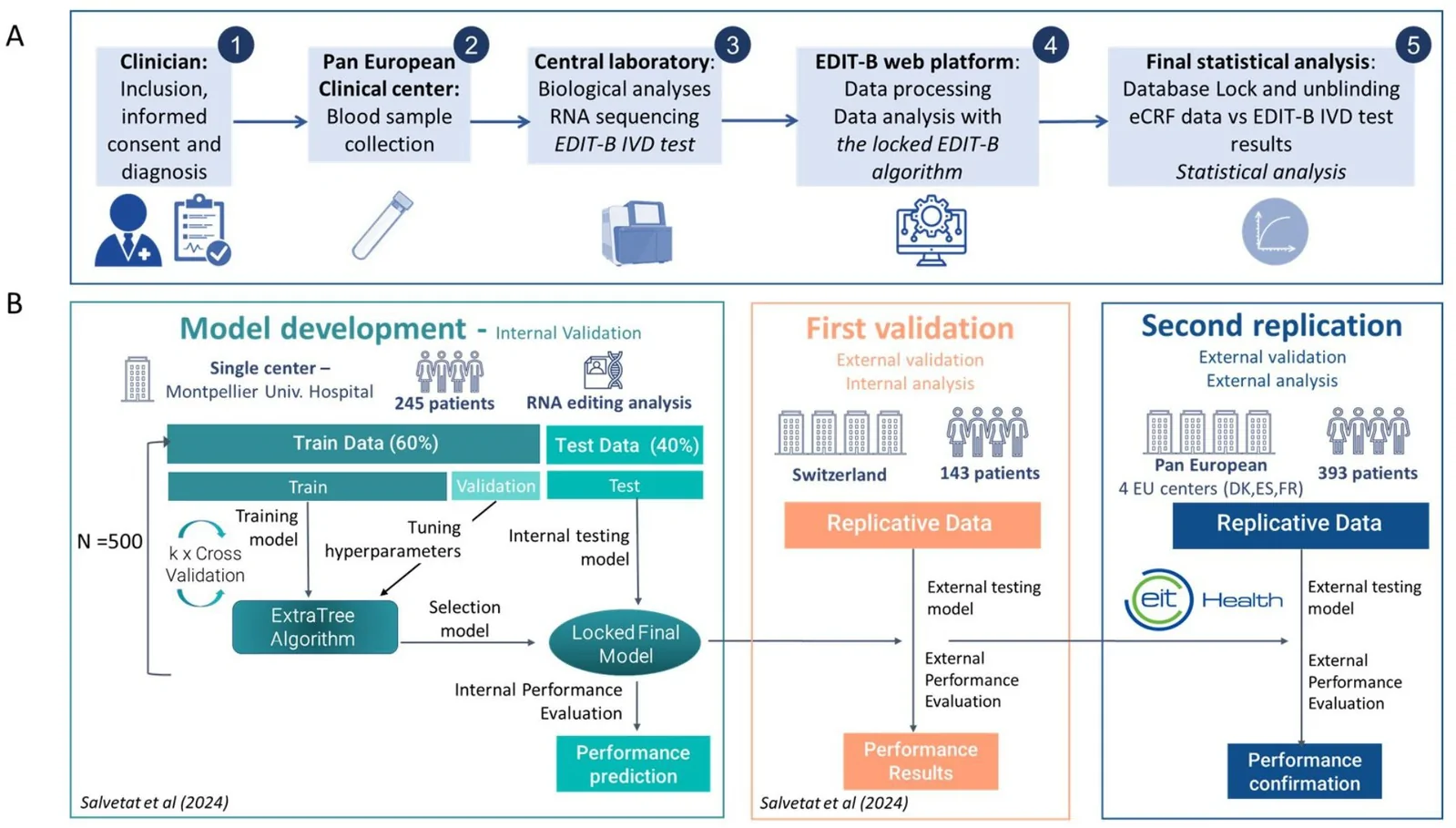
Workflow of the EDIT-B consortium study: from patient recruitment to AI algorithm validation. (A) (1) Participants were enrolled by the clinician and received a diagnostic evaluation. (2) After inclusion, a blood sample was collected at the respective study centre. (3) Samples were shipped to the central laboratory (SYNLAB Italia) to perform the EDIT-B IVD test. (4) Data were analysed using the locked EDIT-B algorithm via the HDS (French Health Data Hosting) EDIT-B web platform. (5) Clinical eCRF data and EDIT-B IVD test results were compared in the final statistical analysis of the locked and certified analytical set. (B) The EDIT-B algorithm was developed by randomly splitting the model development dataset into 60% training and 40% testing subsets. Preprocessing was applied to the training data, and an ExtraTrees model was built using k-fold cross-validation for hyperparameter optimisation. The model was evaluated on the test data, and this process was repeated 500 times with random splits. The final model was selected based on minimising training error and maximising AUC-ROC. It was then locked and applied to two external cohorts (Switzerland study n=143 and pan-European, double-blind, multicentre study n=393) for validation using the same fixed-function parameters as in internal testing. AI, artificial intelligence; AUC, area under the curve; DK, Denmark; eCRF, electronic case report form; ES, Spain; EU, European Union; FR, France; IVD, in vitro diagnostic; ROC, receiver operating characteristic.

Sequencing data were uploaded to the EDIT-B web secure platform, and each patient was processed individually on a health data hosting-certified server using bioinformatic protocol previously detailed.^9^ A standardised report with the EDIT-B result was produced for each patient, including sample identifiers, quality control metrics and algorithm specifications (figure 1A). EDIT-B is a locked ML algorithm with a fixed function based on ExtraTrees method using 22 RNA editing biomarkers, demographic data (age, sex) and psychiatric treatments (antidepressants, antiepileptics, antipsychotics (including lithium), anxiolytics and hypnotics/sedatives, as well as alcohol and tobacco use). Medication was coded according to the WHO Anatomical Therapeutic Chemical (ATC) classification system. The initial development, training and internal validation of the EDIT-B model are shown in figure 1B. The same fixed-function parameters applied to the internal validation were employed in the two external evaluations, ensuring consistency among the cohorts. A description of the artificial intelligence (AI) model development and the associated parameters is detailed in the online supplemental methods and online supplemental table 2.

### Data analysis

Performance of the test was estimated by sensitivity, specificity and overall accuracy. Receiver operating characteristic (ROC) curves and DeLong’s test (pROC R package^12^) were used to evaluate binary classification performance and compare area under the curve (AUC)-ROC values across populations. Precision-recall (PR) curves and the corresponding AUC (AUC-PR) were calculated using the ‘precrec’ R package^13^ to evaluate the model’s ability to identify positive instances while minimising false positives. An optimised cut-off for positive predictive value (PPV), determined in the previous cohort using the cutpointr R package^14^ to minimise false positives, was applied to the current study population. To ensure robustness and generalisability, comprehensive stratified sensitivity analyses were performed on the pooled sample from all centres. These evaluations encompassed psychiatric treatment status (figure 3), clinical complexity and comorbidities (online supplemental figures 9 and 10), demographic and basic clinical characteristics including age and disease onset (online supplemental figures 11–13), disease progression and recurrence markers such as illness duration and depressive episode frequency (online supplemental figures 14–16) and blood collection timing (online supplemental figure 17). Any site-specific harmonisation was performed. Variable importance was assessed in EDIT-B model using TreeSHAP.^15^ TreeSHAP decomposes predictions into additive feature contributions, quantifying each predictor’s impact on the model output. Global importance was computed as the mean absolute Shapley value across observations, reflecting each variable’s overall contribution. Descriptive statistics summarised demographic and clinical characteristics, and effect sizes were calculated using the effectsize R package,^16^ with two-sided 95% CIs reported for all estimates. Model calibration, Target Editing Index (TEI) methods and other additional analysis are described in the online supplemental methods.

## Results

### Patient demographic and clinical characteristics

Patients were matched for sex, age and severity of depression based on Montgomery-Åsberg Depression Rating Scale (MADRS) score, with no significant between-group differences. The proportion of female patients was similar between the MDD and BD groups (61.8% vs 61.3%, respectively, p=1). The great majority of patients (90.8%) were identified as white. No significant differences in ethnic distribution were observed between patients with MDD and those with BD. Body mass index (BMI) was comparable across groups. As expected, depression severity assessed by MADRS score was not statistically significantly different (p=0.9) between both groups (mean scores of 32.1 for patients with MDD and 32.2 for patients with BD). In contrast, patients with BD had statistically significantly higher Young Mania Rating Scale (YMRS) scores than patients with MDD (p<0.001), even their YMRS score stays very low (1.1 for the MDD vs 1.9 for the BD). Regarding psychotropic treatments, approximately 79% of patients received antidepressants, 63% antipsychotics, 35% anxiolytics, 21% hypnotics/sedatives and 35% antiepileptics. Smoking was reported by 37% of patients and alcohol abuse by 8%, with no significant between-group differences (p=1) (table 1). Descriptive statistics, including sample sizes and depression severity, were also performed by recruitment sites (online supplemental table 3).

**Table 1:** Demographic and clinical characteristics of patients included in the analytical set

	Analytical set cohort				
	Total	MDD	BD	Effect size	P value
n	393	238	155		
**Demographic characteristics**
Age					
Min-max	18–79	18–79	18–79		
Mean±SD	46.2±15.3	46.9±15.0	45.1±15.8	0.12* (−0.08, 0.32)	0.2
Sex					
Male, n (%)	151 (38.4)	91 (38.2)	60 (38.7)	1.02† (0.58, 1.81)	1
Female, n (%)	242 (61.6)	147 (61.8)	95 (61.3)		
Race					
Asian, n (%)	6 (1.5)	3 (1.3)	3 (1.9)		
Black, n (%)	13 (3.3)	7 (2.9)	6 (3.9)		
White/Caucasian, n (%)	357 (90.8)	216 (90.8)	141 (91.0)		
Other, n (%)	17 (4.3)	12 (5.0)	5 (3.2)		
All				0.06‡ (0.00, 0.12)	0.7
BMI, kg/m^2^ (mean±SEM)	26.2 (±5.5)	26.2 (±5.6)	26.1 (±5.3)	0.02* (−0.18, 0.22)	0.9
**Clinical characteristics**					
Symptom severity scores
MADRS score (mean±SEM)	32.1 (±6.5)	32.1 (±6.6)	32.2 (±6.3)	−0.02* (−0.22, 0.19)	0.9
YMRS score (mean±SEM)	1.4 (±2.0)	1.1 (±1.8)	1.9 (± 2.3)	−0.40* (−0.60, −0.19)	**<0.001**
Psychotropic treatments					
Anxiolytics, n (%)	139 (35.4)	100 (42.0)	39 (25.2)	0.47† (0.26, 0.85)	**<0.05**
Hypnotics/sedatives, n (%)	83 (21.1)	58 (24.4)	25 (16.1)	0.59† (0.29, 1.20)	0.2
Antiepileptics, n (%)	136 (34.6)	41 (17.2)	95 (61.3)	7.63† (3.95, 14.71)	**<0.001**
Antipsychotics, n (%)	249 (63.4)	115 (48.3)	134 (86.5)	6.86† (3.42, 13.74)	**<0.01**
Antidepressants, n (%)	309 (78.6)	232 (97.5)	77 (49.7)	0.03† (0.01, 0.09)	**<0.001**
All treatment classes				0.35‡ (0.25, 0.43)	0.2
Substance addiction					
Tobacco, n (%)	147 (37.4)	88 (37.0)	59 (38.1)		
Alcohol, n (%)	30 (7.6)	19 (8.0)	11 (7.1)		
All				0.03‡ (0.00, 0.22)	1

Data are the mean±SEM. P values of main characteristics are obtained with the Student’s t-test or χ2 test.

Bold values indicate p<0.05

* Cohen’s d. Benchmarks: |d| ≈0.2 small, ≈0.5 medium, ≈0.8 large.

† OR.

‡ Cramér’s V. Benchmarks: ≈0.1 small, ≈0.3 medium, ≈0.5 large.

BD, bipolar disorder; BMI, body mass index; MADRS, Montgomery-Åsberg Depression Rating Scale; MDD, major depressive disorder; YMRS, Young Mania Rating Scale.

Regarding sociodemographic characteristics, participants were commonly single (42.2%), followed by married (32.1%) and separated/divorced (22.9%). Most lived alone (36.6%), with a spouse/partner (35.6%) or with children (26.2%). Over half reported a disability, predominantly temporary (37.7%) or permanent (14.0%). Educational attainment was high: 91.1% had completed secondary school and 49.6% held a university degree. No significant differences were observed between MDD and BD groups (online supplemental table 4).

The mean duration of illness was comparable between the two groups (around 10 years). The onset of disease and the first depressive episode tended to occur earlier in patients with BD (p<0.001). Among the patients with BD, the first depressive episode typically preceded the first manic/hypomanic episode. Patients with BD experienced a higher number of depressive episodes compared with patients with MDD. Depressive polarity at onset was predominant in patients with BD (88.4%). Psychotic symptoms were present in approximately one-third of patients with BD, compared with fewer than 10% of patients with MDD. A lifetime history among first or second generations of affective disorders was reported by 72.9% of patients with BD and 49.2% of patients with MDD, while a family history of psychiatric illness was more common in patients with BD (81.3%) than in patients with MDD (60.5%). A family history among first or second generations of completed suicide was reported by approximately 20% of patients in both groups (online supplemental table 5).

According to the Mini-International Neuropsychiatric Interview, all participants fulfilled criteria for an ongoing MDE of at least 2 weeks’ duration, with most reporting a recurrent history. Manic or hypomanic episodes were absent in the MDD group, whereas 40.0% and 63.9% of patients with BD reported lifetime manic and hypomanic episodes, respectively. Recent suicide attempts were common in both groups (20–30%). Psychiatric comorbidities included panic disorder (19.1% overall; 26.5% in BD vs 14.3% in MDD), generalised anxiety disorder (15.0%, similar across groups), psychotic disorders (3.8%, predominantly in BD type I (BD-I)), alcohol use disorder (7.6%), substance use disorders (4.3%), obsessive-compulsive disorder (8.4%) and eating disorders (2.0%) (online supplemental table 6).

### EDIT-B test performances

The EDIT-B test was evaluated in this study (n=393) (figure 2A). The empirical ROC curve presents an AUC-ROC of 0.873 (95% CI 0.837 to 0.909) and an AUC-PR of 0.843 (95% CI 0.798 to 0.884; online supplemental figure 3A) for EDIT-B test. The 95% CIs for the AUC-ROC of EDIT-B test overlapped with those previously reported for the Swiss external validation (AUC-ROC 0.904, 95% CI 0.853 to 0.955) (figure 2B), confirming statistical consistency (DeLong’s test, p=0.33).

**Figure 2:**
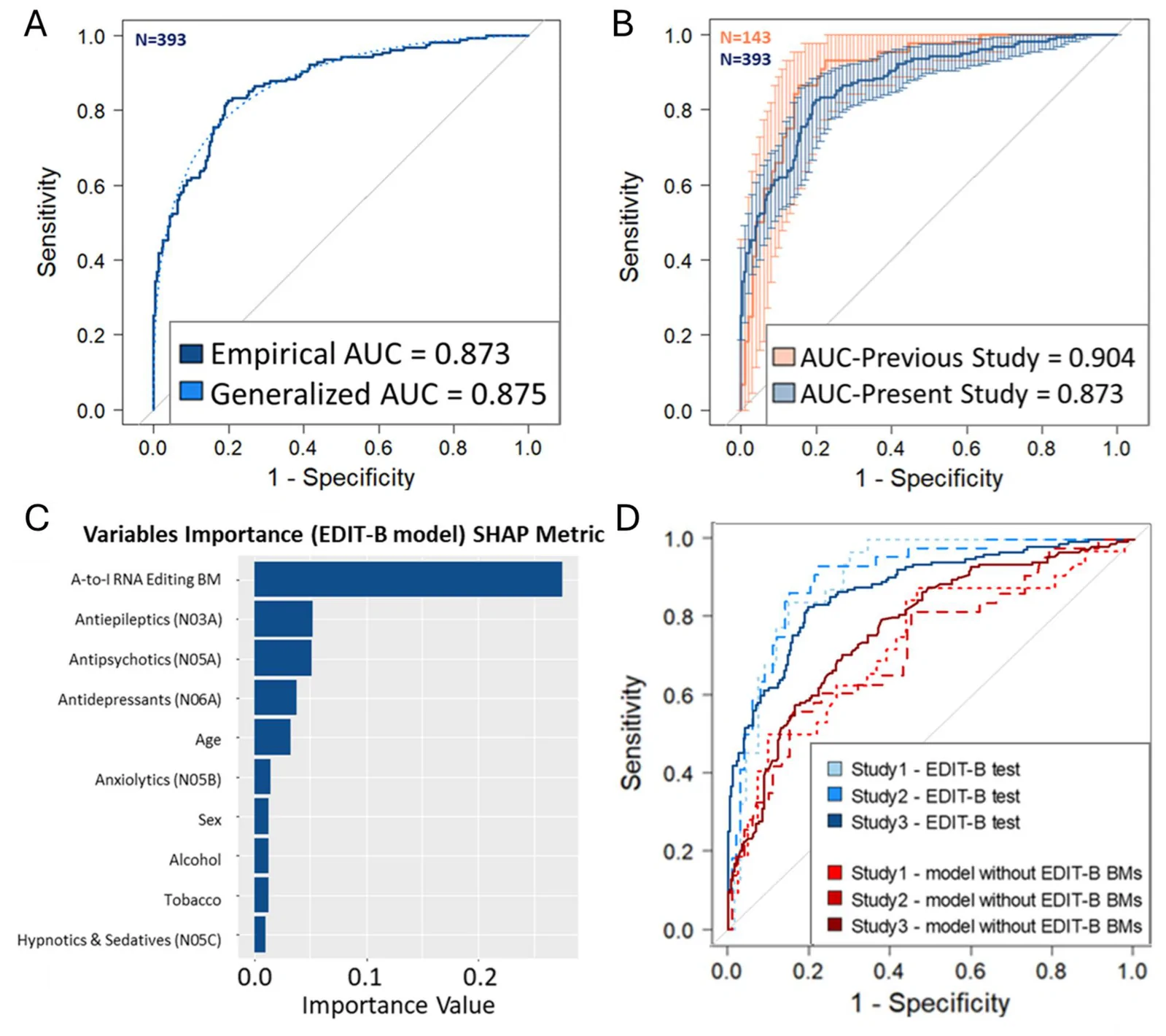
Diagnostic performances, explicability and impact of RNA editing BMs of EDIT-B test. (**A**) ROC curves of EDIT-B test in the present study (analytical set, n=393): light blue line—generalised area under the ROC curve; dark blue line—empirical area under the ROC curve. (**B**) Comparison of the ROC curves and CIs of EDIT-B test in the present study versus previous results^9^: Corail line—previous study ROC curve (Salvetat *et al*, n=143); dark blue line—present study ROC curve (analytical set, n=393). (**C**) Variable importance using SHAP metric: bar plot illustrates the importance of variables of mean absolute Shapley values in the EDIT-B test. The length of each bar indicates the relative contribution of each variable to the model’s predictions. The variable ‘A-to-I RNA Editing BM’ exhibits the highest importance, followed by ‘Antiepileptics (N03A)’, ‘Antipsychotics (N05A)’ and other factors. This analysis helps identify the key drivers of the model’s performance. (**D**) EDIT-B model development and comparison to clinical variables model excluding EDIT-B BMs. ROC curves were generated to illustrate and compare the diagnostic performance of two models, one including RNA editing BMs (EDIT-B test) and one excluding them across the same three cohorts (development cohort: study 1 (n=98); Switzerland cohort: study 2 (n=143); current pan-European, multicentre cohort: study 3 (n=393)). The inclusion of EDIT-B RNA editing BMs statistically improves model performance (DeLong’s test, p<0.05), as reflected by higher AUC-ROC values in the blue curves (respectively AUC study 1=0.901, AUC study 2=0.904 and AUC study 3=0.873) compared with the red curves (respectively AUC study 1=0.732, AUC study 2=0.723 and AUC study 3=0.772). A-to-I, adenosine-to-inosine; AUC, area under the curve; BMs, biomarkers; ROC, receiver operating characteristic; SHAP, SHapley Additive exPlanations.

False-positive (patients with MDD classified as BD by EDIT-B) and false-negative (patients with BD classified as MDD by EDIT-B) cases were also characterised (online supplemental table 7). No significant differences were observed in age at onset, illness duration, psychotic symptoms or frequency of depressive episodes (all p>0.2). Except for alcohol use disorder and the number of mania episodes (p<0.01), psychiatric comorbidities showed no significant association with misclassification (p>0.2).

The diagnostic performances for EDIT-B test (online supplemental table 8) achieved a sensitivity of 82.6% (95% CI 75.7% to 88.2%), a specificity of 80.3% (95% CI 74.6% to 85.1%), a negative predictive value of 87.6% (95% CI 82.5% to 91.7%), a PPV of 73.1% (95% CI 65.9% to 79.6%) and an overall accuracy of 81.2% (95% CI 77.0% to 84.9%). These results confirm the potential use of the EDIT-B test for providing a robust separation between patients with MDD and BD.

Among the four recruiting centres involved in this study, three were BD reference centres (Centre 001, Spain; Centre 002, Denmark; Centre 003, France). BD reference centres are recognised for their expertise in the management of BD but also treat a broad psychiatric population, including patients with MDD. A detailed centre-based analysis was conducted to assess the AUC-ROC and AUC-PR curves of the EDIT-B test across the three BD reference centres (online supplemental figures 4A–C and 5A–C). Overall, the EDIT-B test showed high performance across all three BD reference centres (AUC-ROCs of 0.898 (Centre 001), 0.887 (Centre 002) and 0.893 (Centre 003), respectively; AUC-PRs of 0.868 (Centre 001), 0.900 (Centre 002) and 0.893 (Centre 003), respectively). Notably, AUC-ROC curves measured in the BD reference centres were superior to the non-BD reference centre (Centre 004, Spain; AUC 0.844). When combining data from the three BD reference centres (n=295), the EDIT-B test achieved an AUC-ROC of 0.891 and an AUC-PR of 0.892 (95% CI 0.855 to 0.927) (online supplemental figures 3B and 6A, online supplemental table 8) without significant differences from previously^9^ reported performance (DeLong’s test, p=0.7; online supplemental figure 6B).

### EDIT-B model assessment

#### A-to-I RNA editing biomarkers

The panel of RNA editing-based blood biomarkers detailed above was sequenced in the current dataset (n=393). Differential analysis of selected RNA editing biomarkers was summarised in a ‘Target Editing Index’ (TEI) by gene, using the same method and preprocessing as the one applied to the development cohort (initial cohort used to develop and calibrate the EDIT-B algorithm prior to independent validation), showing highly significant discrimination (adjusted p<0.05) between MDD and BD populations, demonstrating robust biomarker performance across cohorts (online supplemental figure 7A–D).

#### Calibration and predictive density

We further assessed the reliability of the probabilistic predictions of EDIT-B using the analytical dataset (n=393). EDIT-B demonstrated robust calibration and predictive performance across the cohort (Brier score=0.19, expected calibration error=0.19). The observed agreement between the predicted probabilities and the actual observed outcomes is illustrated in the density and calibration plots (online supplemental figure 8A,B).

#### Feature importance and sensitivity analysis

A global sensitivity analysis was performed to quantify the relative contribution of the variables to diagnostic predictions. SHapley Additive exPlanations (SHAP) analysis identified biomarker-related variables as the primary drivers of model predictions compared with treatment variables and other clinical factors (age, sex and addictions; figure 2C).

To assess the impact of RNA editing biomarkers on the diagnosis performance, the EDIT-B model was compared with a similar model not using RNA editing biomarkers. Briefly, in this analysis we compared: (a) a model without the RNA editing biomarkers including only clinical factors such as age, sex, addiction and psychiatric treatment variables, and (b) EDIT-B, which integrates RNA editing biomarkers with clinical factors and psychiatric treatment features. Both models followed the same preprocessing and ML framework across the same three cohorts (development cohort: study 1; first Swiss external validation cohort: study 2; current second European external validation cohort: study 3), with discrimination assessed via AUC-ROC as the primary performance metric. The EDIT-B model achieved the highest AUC-ROC and significantly outperformed the model with age, sex, addictions and treatment alone in the three different cohorts (DeLong’s test, p<0.05 for three comparisons) (figure 2D).

Further, to assess a putative confounder effect of medication on EDIT-B performances, a detailed stratified analysis according to psychiatric treatment classes (based on WHO ATC) and patient status (treated vs untreated) is provided in figure 3; briefly, EDIT-B algorithm performance exhibits no significant differences in the different cases (treatment with antidepressants, antipsychotics, anxiolytics and hypnotics/sedatives) except for antiepileptic treatment, for which diagnostic performances (AUC) were higher in untreated patients. The performance of EDIT-B was also evaluated across age, sex and BD subtypes, and a detailed subgroup analysis is provided in online supplemental figures 11 and 12 and online supplemental table 9. In summary, algorithm performance was consistent across disease characteristics, psychiatric comorbidities, sex and age groups, while a higher AUC was observed in BD-I compared with BD type II (BD-II), although both subtypes maintained high diagnostic accuracy (>80%).

**Figure 3:**
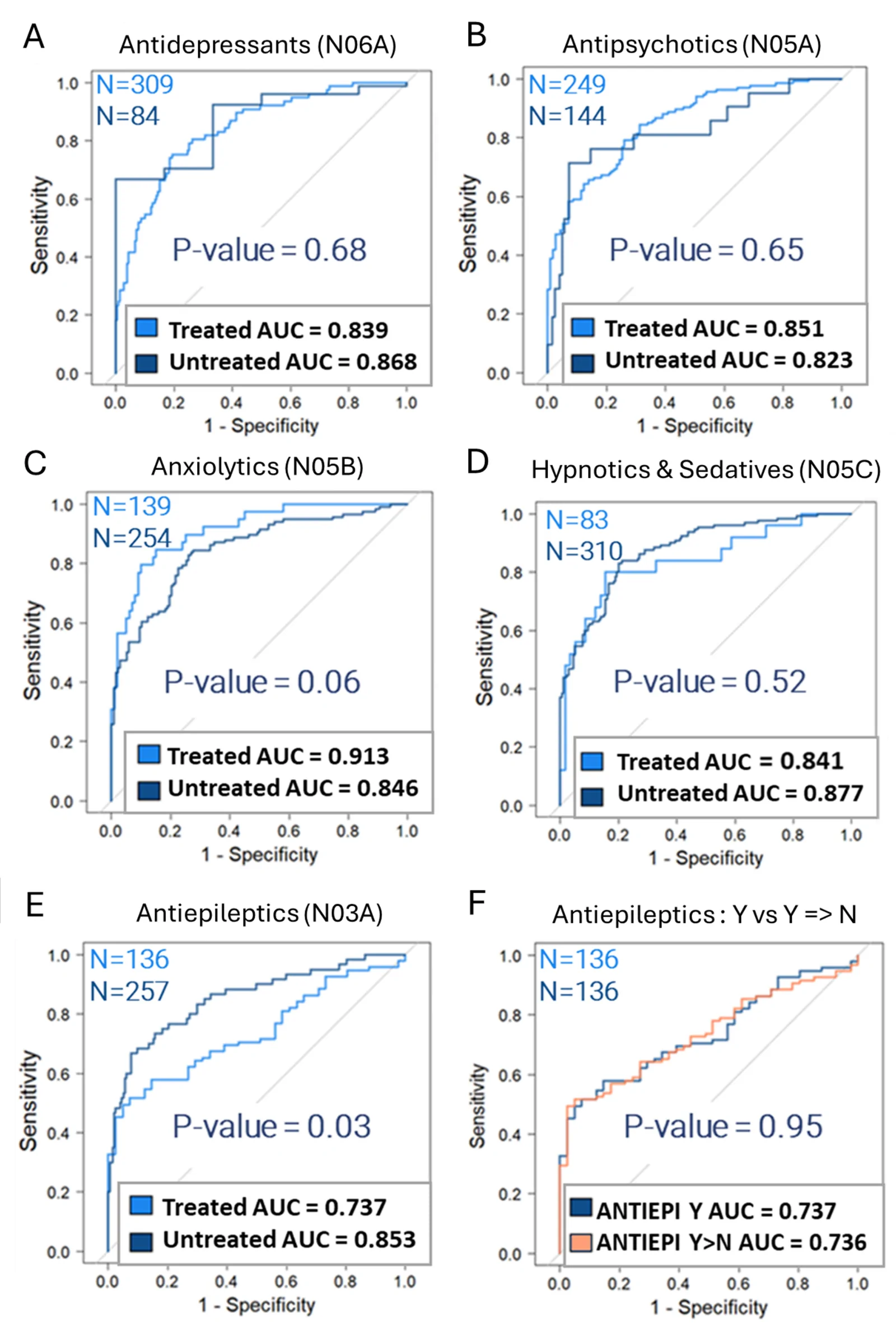
Stratified sensitivity analysis according to psychiatric treatment status. To evaluate the potential medication-related confounding effect, we conducted a stratified sensitivity analysis according to psychiatric treatment status (treated vs untreated) for (A) antidepressant treatment (N06A; treated=309 and untreated=84 patients), (B) antipsychotic treatment (N05A; treated=249 and untreated=144 patients), (C) antiepileptic treatment (N03A; treated=136 and untreated=257 patients), (D) anxiolytic treatment (N05B; treated=139 and untreated=254 patients) and (E) hypnotic/sedative treatment (N05C; treated=83 and untreated=310 patients). ROC curves and corresponding AUC values for treated and untreated patients across different psychiatric medication classes are displayed for this analysis, along with the p values indicating the statistical significance of the differences between the two groups. Notably, the comparison of AUC values for antiepileptics (DeLong’s test, p=0.03) is the only one showing a statistically significant difference. (F) The comparison between real antiepileptic drug uptake (antiepileptic-treated patients) and simulated no antiepileptic drug uptake (the model is artificially instructed to consider that patients are not taking antiepileptics) demonstrates no significant difference (p=0.95), supporting the robustness of the findings and indicating consistent diagnostic accuracy irrespective of treatment exposure. Psychiatric treatments were classified according to the WHO ATC classification system. ATC, Anatomical Therapeutic Chemical; AUC, area under the curve; ROC, receiver operating characteristic.

Additionally, diagnostic accuracy remained consistently high in younger populations (aged 18–25, 18–30 and 18–35; AUCs: 0.902, 0.917 and 0.877, respectively; all p>0.35) (online supplemental figure 12) and remained consistent regardless of the age of disease onset (≤20 or ≤26 years; p=0.90 or 0.76, respectively) (online supplemental figure 13). Similarly, discriminating power remained stable across illness duration (including early stages ≤1 year; AUC=0.893) (online supplemental figure 14) and regardless of the number of depressive episodes (≤3 or ≤4 episodes; p=0.71 or 0.35) (online supplemental figure 15). Finally, high sensitivity was maintained independently of the frequencies of manic, hypomanic and mixed episodes, with false-negative rates decreasing as episode frequency increased (online supplemental figure 16). Blood collection timing was homogeneous along the day for both groups, with no significant impact on the performances of the EDIT-B test (p=0.33) (online supplemental figure 17). Together, these findings corroborate the robustness of EDIT-B across diverse clinical trajectories and disease characteristics.

Pearson correlation analyses were performed between the RNA editing levels of biomarkers and various clinical, demographic and treatment-related covariates. A moderate association (0.2–0.4) was observed between the number of episodes (manic, hypomanic and mixed), the age at onset of mania and RNA editing biomarkers from the genes *GAB2, LYN, PTPRC, MDM2, PRKCB and ZNF267* (online supplemental figure 18).

#### Diagnostic utility

To evaluate the clinical utility of the model, a decision curve analysis was performed using the analytical dataset. In brief, the model demonstrated a higher net benefit compared with the default strategies of classifying all patients as bipolar or classifying none as bipolar across a wide range of threshold probabilities (online supplemental figure 19).

## Discussion

This study demonstrates that the innovative EDIT-B test has the potential to serve as a complementary tool to clinical evaluation and to improve the accuracy of differential diagnosis between MDD and BD. If implemented in clinical routine, such a tool could reduce the diagnostic delay for BD, enabling earlier patient management and appropriately tailored treatment, reducing the risk of antidepressant-induced mania^17^ and ultimately lowering hospitalisation rates and associated healthcare costs. Taken together, these results position the EDIT-B test as a potentially clinically actionable innovation with direct translational value for personalised and precision psychiatry.

Previous publication demonstrated that using EDIT-B allows differentiation of MDD from BD in two independent cohorts recruited in France and Switzerland. In the present study, we extend this work by evaluating the EDIT-B test (consisting of a locked model using exactly the same panel and algorithm/parameters) in a third, large and independent European multicentre cohort spanning three countries (France, Spain and Denmark). The current study validated EDIT-B test performances, combining high discriminative performance (accuracy, sensitivity and specificity >80%) with strong reproducibility across diverse clinical settings. When performed on the 393 patients from four centres in three countries, EDIT-B closely replicated the previous results from the original validation study,^9^ underscoring its robustness across independent cohorts. Moreover, the observed performances were comparable in the great majority of stratification analyses, including psychiatric treatment, age at onset, illness duration, number of depressive/manic episodes, comorbidities, disease characteristics, BD subtype and sex. Remarkably, age-stratified analyses demonstrate diagnostic performance consistent across all age groups. This finding suggests the potential of the test for early clinical implementation. Altogether, these data support both EDIT-B clinical reliability and its potential for broad generalisability in real-world practice.

When analysis was restricted to patients clinically diagnosed at BD reference centres, EDIT-B performance was greater than that observed for the total population. There is a slight difference in concordance between the specialised reference centres for BD and the general psychiatry centre. Indeed, a lower PPV indicates a higher proportion of false positives in this centre, which slightly reduces the overall accuracy of the model. It is crucial that the diagnosis established by clinicians be as reliable as possible, since this test compares the clinical diagnosis with the EDIT-B test. This finding highlights the usefulness of such a tool as a diagnostic aid, particularly in general psychiatry centres.

Regarding the study population, participants with MDD and BD exhibited largely comparable baseline clinical characteristics, including age, sex, race, BMI, psychiatric treatment, suicidality, suicide behaviour and comorbidities, with the exception of a higher prevalence of panic disorders in BD. This consistency confirms that the recruitment process rigorously adhered to predefined inclusion criteria, ensuring balanced group comparisons. Moreover, while the mean duration of illness was comparable across groups, BD tended to manifest earlier, with the first depressive episode typically preceding the onset of mania or hypomania. In this line, patients with BD experienced a higher lifetime number of depressive episodes as compared with patients with MDD.^18^ Family history patterns also revealed notable differences. A psychiatric family history was more prevalent in BD than in MDD, confirming the strong heritable component of BD, reinforcing its genetic underpinnings. Taken together, these findings underscore the complexity of mood disorders and the challenges of differentiating BD from MDD based solely on clinical features during MDE.

Based on clinical features, distinguishing an MDE as part of BD versus MDD remains relatively subjective. This contributes to delays in accurate diagnosis, with the clinician’s experience and expertise playing a crucial role in improving diagnostic accuracy. In primary care, the Mood Disorder Questionnaire has demonstrated a sensitivity around 70% in psychiatric outpatients^19^ and good accuracy for screening BD. In the same way, some studies indicate that for patients with bipolar spectrum illness, the sensitivity reaches 70% only for BD-I.^20^ Meanwhile, scales such as the 32-item Hypomania Symptom Checklist, First Revision, have shown utility in detecting hypomanic symptoms,^21^ which can further aid in identifying at-risk patients. This confirms the need for an additional tool for the differential diagnosis of BD and MDD.

The biological plausibility of the EDIT-B signature is supported by the convergence of its constituent genes on pathways involved in immune signalling, neuroinflammation, oxidative stress and mitochondrial dysfunction. Notably, *IFNAR1*, *IL17RA*, *PTPRC* (also known as *CD45*), *LYN*, *GAB2* and *PRKCB* participate in interconnected signalling networks regulating microglial activation and inflammatory responses through the PI3K-AKT, NF-κB and MAPK pathways,^22–26^ while *MDM2* is associated with oxidative stress and mitochondrial homeostasis via the AKT-p53 axis.^27 28^ The relationship between *ZNF267* and the proposed neuroimmune signalling axis remains more elusive. As a transcription factor, *ZNF267* may modulate the expression of genes related to mood disorder and neuroinflammatory responses.^29^ Together, these findings support the hypothesis that the EDIT-B signature reflects coordinated neuroimmune and oxidative stress mechanisms implicated in mood disorders.

EDIT-B puts on evidence the potential use of molecular signatures to elucidate the biological underpinnings of depression and BD. In this sense, feature importance and sensitivity analyses presented here highlight the key contribution of RNA editing biomarkers to BD diagnosis. The integration of RNA sequencing and ML is emerging as a powerful approach to enhance diagnostic precision in medicine, enabling the identification of robust disease-specific molecular signatures with high precision. This approach underscores the transformative potential of AI-driven genomics and epigenomics in improving clinical outcomes, including for complex disorders in psychiatry, where epitranscriptomic signatures such as the EDIT-B test show great promise.

### Limitations

The present study has some limitations. First, the majority of participants were white/Caucasian, reflecting the demographic profile of the recruitment centres. It may limit the generalisability of our findings to more diverse populations. Second, the patients enrolled in this study were moderately or severely depressed, limiting the interpretation of EDIT-B results for the low severity depression states. Regarding the different bipolar subclassifications (ie, BD-I vs BD-II), diagnostic performance varied between BD-I and BD-II in distinguishing MDD from BD. Nonetheless, both subtypes achieved high diagnostic accuracy (>80%). Third, while EDIT-B results demonstrated no significant differences in most stratified sensitivity analyses across medication subgroups, a significant difference was observed in the analysis of patients treated with antiepileptic drugs. However, the comparison between real antiepileptic drug uptake in treated patients and simulated no antiepileptic drug uptake (in which the model was artificially instructed to consider the patients were not taking antiepileptics) showed no significant difference (p=0.95). Fourth, the relatively high cost of the NGS (Next-Generation Sequencing)-based test may restrict its availability in clinical settings. Fifth, due to the real-world design of the study, detailed medication timing and fasting status were not recorded; however, subgroup analysis by sampling time suggests that the EDIT-B test performance is stable across the day. Finally, the study was prospective but cross-sectional, although based on patients with a proven BD or MDD trajectory; a longitudinal study with first-episode major depression would provide the greatest evidence in favour of the predictive value of the test.^30^ To this end, the generalisability to high-genetic-risk populations is currently being addressed in an ongoing prospective study (NCT06507787).

## Conclusion

In summary, this study provides additional evidence supporting the validity and clinical performance of the EDIT-B test as a potential complement to the objective diagnosis of BD in patients with depression. These findings contribute to the advancement of precision psychiatry and suggest that such molecular biomarkers could help reduce misdiagnosis and the risk of inappropriate treatment in clinical practice.

## Supplementary material

10.1136/bmjment-2026-302859 online supplemental file 1

10.1136/bmjment-2026-302859 online supplemental file 2

## Data Availability

No data are available.
