# Supplementary material for: EDIT-B consortium: an external pan-European validation of an innovative machine learning-based test for bipolar disorder diagnosis
Source: BMJ Ment Health. 2026 Oct 5;29(1):e302859. doi: 10.1136/bmjment-2026-302859 (PMC13646935; doi:10.1136/bmjment-2026-302859)
Supplement: Supplementary file 2 [file bmjment-29-1-s002.pdf]

“Clinical validation study for EDIT-B test: an aid for differential diagnosis of bipolar disorder, based on RNA editing blood biomarkers”

ACRONYM: **EDIT-B**

CLINICAL PERFORMANCE STUDY PROTOCOL
CONCERNING AN IN VITRO DIAGNOSTIC MEDICAL DEVICE

Version no. 3.0 dated 15/05/2024
Project code: NA

Coordinating Investigator: Pr Eduard Vieta
Hospital Clinic de Barcelona
Villarroel, 170
08036 Barcelona, Spain
Tel: +34-93-227-5477
Fax: +34-93-227-9228
E-mail: evieta@clinic.cat

Scientific Director: Dinah Weissmann
Cap Gamma
1682 rue de la Valsière
34184 Montpellier, France
Tel: +33 4 67 04 74 64
E-mail: dweissmann@alcediag-alcen.com

Sponsor : Alcediag
Cap Gamma
1682 rue de la Valsière
34184 Montpellier, France
Tel: +33 4 67 04 74 64
E-mail: dweissmann@alcediag-alcen.com
www.alcediag-alcen.com

Entity responsible for monitoring the study, data management and data analysis: AIXIAL
40 Avenue André Morizet
92100 Boulogne-Billancourt, France
Tel: +33 6 71 09 82 56
E-mail: louise.goedhart@aixial.com
www.aixial.com

Central Laboratory: Synlab Italia – Genetic Unit
via Beato L. Pavoni 18
25014 Castenedolo (BS), Italy

Revision	Date	Revision History
1.0	December 28th, 2021	Initial version
2.0	February 25th, 2022	Modification of paragraphs *10.1 Inclusion and last study visit* and *20 Informed consent process* :<br>Deletion of the study subject's reflection time of at least 30 minutes
2.1	November 15th, 2022	Modification of<br>- Paragraph *7.5.2: Identification of participants*:<br>Deletion of patients initials for patient ID.<br>- Paragraph *9.6.1: Blood samples* and *11.1 Sample collection and processing*:<br>Addition of a paragraph explaining blood sample transfer to another Party.<br>- Paragraph *10.1: Inclusion and last study visit*:<br>Addition of explication that the questionnaire of life will be used twice: for the current situation and the time before correct diagnosis.<br>- Paragraph *21.2 The role of the investigator*:<br>Deletion of SAE recording in e-CRF.
3.0	May 15th, 2024	Modification of<br>- Paragraphs *2 Study summary, 9.5 Number of subjects* and *10.2 Duration of the study:*<br>Modification of study duration (32 months instead of 24 months).<br>- Paragraph *9.5 Number of subjects:*<br>Modification of enrolment number/month/centre (3.5 instead of 4.5).<br>Addition of a note mentioning competitive enrolment.<br>- Paragraphs *2 Study summary* and *7.5.1 Design of the study*<br>Modification of population ratio (up to 2:1 instead of fixed 1:1) in the paragraphs.<br>- Paragraphs *2 Study summary* and *11.2 Procedures associated with the use of the device*<br>Completion of individual data information used for the test.

SIGNATURE page for a research PROTOCOL

Research code number:

Title: "Clinical validation study for EDIT-B test: an aid for differential diagnosis of bipolar disorder, based on RNA editing blood biomarkers"

Version no. 3.0 dated: 15 / 05 / 2024

The study will be carried out in accordance with the protocol, with current good practices and with statutory and regulatory requirements.

Coordinating Investigator:
Professor Eduard Vieta
Hospital Clinic de Barcelona
Villarroel, 170
08036 Barcelona, Spain

Date: 29/05/24
Signature: Eduard Vieta (May 29, 2024 12:24 GMT+2)

Sponsor
Alcediag
Cap Gamma
1682 rue de la Valsière
34184 Montpellier, France

Date: 29/05/24
Signature: Dinah WEISSMANN

TABLE OF CONTENTS

1 GLOSSARY 7

2 STUDY SUMMARY 8

3 SCIENTIFIC JUSTIFICATION FOR THE STUDY 14

3.1 HYPOTHESIS FOR THE STUDY 14
3.2 DESCRIPTION OF KNOWLEDGE RELATING TO THE CONDITION IN QUESTION 14
3.3 SUMMARY OF RELEVANT PRE-CLINICAL EXPERIMENTS AND CLINICAL TRIALS 14

4 IVD MEDICAL DEVICE UNDER INVESTIGATION 16

4.1 SUMMARY DESCRIPTION OF THE IVD MEDICAL DEVICE UNDER INVESTIGATION 16
4.2 IDENTIFICATION AND INSTRUCTIONS FOR USE 16
4.2.1 Name of the IVD medical device 16
4.2.2 Intended purpose of the device 16
4.3 REQUIRED TRAINING AND EXPERIENCE 16

5 SPONSOR 16

6 STUDY SITES 17

6.1 PRINCIPAL INVESTIGATOR RESPONSIBILITIES 17

7 OBJECTIVES OF THE CPSP 17

7.1 STUDY OBJECTIVE 17
7.2 ENDPOINT 17
7.3 CLAIMS AND INTENDED PERFORMANCE OF THE IVD MEDICAL DEVICE UNDER INVESTIGATION THAT ARE TO BE EVALUATED 18
7.4 RISKS AND BENEFITS 18
7.5 DESCRIPTION OF RESEARCH METHODOLOGY 18
7.5.1 Design of the study 18
7.5.2 Identification of participants 18

8 IVD COMPARATOR 18

9 STUDY POPULATION AND SPECIMENS 18

9.1 VALIDATED SPECIMEN TYPE 18
9.2 INCLUSION CRITERIA 19
9.3 EXCLUSION CRITERIA 19
9.4 INFORMATION NECESSARY TO CHARACTERISE THE SUBJECT 19
9.5 NUMBER OF SUBJECTS 20
9.6 SPECIMEN STORAGE, HANDLING, TRANSPORT, AND DISPOSAL 20
9.6.1 Blood samples 20
9.6.2 Biological samples collection 20

10 IMPLEMENTATION OF THE STUDY 21

10.1 INCLUSION AND LAST STUDY VISIT 21
10.2 DURATION OF THE STUDY. 23
10.3 DISTINCTION BETWEEN STANDARD CARE AND STUDY 23
10.4 TERMINATION RULES 23
10.4.1 Criteria and procedure for premature withdrawal of a participant from the study 23
10.4.2 Follow-up of participants following premature withdrawal from the study 23
10.4.3 Procedures for replacing participants, if applicable 23
10.4.4 Full or partial discontinuation of the study 24

11 STUDY PROCEDURES 24

11.1 SAMPLE COLLECTION AND PROCESSING 24
11.2 PROCEDURES ASSOCIATED WITH USE OF THE DEVICE 24
11.3 DESCRIPTION OF PERFORMANCE OR EFFICACY ASSESSMENT PARAMETERS 25

12 MONITORING PLAN 25

12.1 QUALITY ASSURANCE 25

12.1.1 General organisation ... *25*
12.1.2 Strategy for centre opening ... *25*
12.1.3 Scope of centre monitoring ... *25*
12.1.4 Quality control ... *26*
12.1.5 Case report forms ... *26*
12.2 AUDITS AND INSPECTIONS ... 26

13 SPECIFIC STUDY COMMITTEES ... 27

13.1 STEERING COMMITTEE ... 27

14 DATA MANAGEMENT ... 27

14.1 DATA COLLECTION PROCEDURES ... 27
14.2 IDENTIFICATION OF DATA RECORDED DIRECTLY IN THE CRFS WHICH WILL BE CONSIDERED AS SOURCE DATA ... 28
14.3 RIGHT TO ACCESS DATA AND SOURCE DOCUMENTS ... 28
14.3.1 Data access ... *28*
14.3.2 Source documents ... *28*
14.3.3 Data confidentiality ... *29*
14.4 DATA PROCESSING AND STORAGE OF RESEARCH DOCUMENTS AND DATA ... 29
14.4.1 Identification of the data processing manager and location(s) ... *29*
14.4.2 Data entry ... *29*
14.4.3 Data ownership ... *29*

15 STATISTICAL CONSIDERATIONS ... 29

15.1 DESCRIPTION OF STATISTICAL METHODS TO BE USED INCLUDING THE TIMETABLE FOR THE PLANNED INTERIM ANALYSES ... 29
15.2 CALCULATION HYPOTHESES FOR THE NUMBER OF PARTICIPANTS REQUIRED AND THE RESULT ... 30
15.3 ANTICIPATED LEVEL OF STATISTICAL SIGNIFICANCE ... 30
15.4 STATISTICAL CRITERIA FOR TERMINATION OF THE STUDY. ... 30
15.5 METHOD FOR TAKING INTO ACCOUNT MISSING, UNUSED OR INVALID DATA ... 30
15.6 MANAGEMENT OF MODIFICATIONS MADE TO THE ANALYSIS PLAN FOR THE INITIAL STRATEGY. ... 31
15.7 CHOICE OF INDIVIDUALS TO BE INCLUDED IN THE ANALYSES ... 31

16 AMENDMENTS TO THE CPSP ... 31

17 DEVIATIONS FROM CLINICAL PERFORMANCE STUDY PROTOCOL ... 31

17.1 DEFINITIONS ... 31
17.2 DEVIATION DETECTION ... 32
17.3 DEVIATION ANALYSIS AND CHOICE OF ACTIONS ... 32
17.4 REPORTING OF MAJOR OR CRITICAL DEVIATIONS ... 32

18 ACCOUNTABILITY OF IVDMD UNDER INVESTIGATION ... 32

19 STATEMENTS OF CONFORMITY ... 33

19.1 COMPLIANCE WITH THE DECLARATION OF HELSINKI ... 33
19.2 REQUEST FOR APPROVAL FROM THE RESEARCH ETHICS COMMITTEE ... 33
19.3 REQUEST FOR AUTHORISATION FROM THE COMPETENT AUTHORITY (FROM EACH PARTICIPATING COUNTRY) ... 33
19.4 PROCEDURES RELATING TO DATA PROTECTION REGULATIONS ... 33

20 INFORMED CONSENT PROCESS ... 33

21 SAFETY ASSESSMENT - RISKS AND BURDEN ADDED BY THE STUDY ... 34

21.1 DEFINITIONS ... 34
21.2 THE ROLE OF THE INVESTIGATOR ... 35
21.2.1 Serious adverse events that require the investigator to notify the sponsor without delay *35*
21.2.2 Specific features of the protocol ... *35*
21.2.3 Period during which SAEs must be notified without delay by the investigator to the sponsor *35*
21.2.4 Procedures and deadlines for notifying the sponsor ... *35*
21.3 ROLE OF THE SPONSOR ... 35

21.3.1 Analysis and declaration of serious adverse events ... 36
21.3.2 Analysis and declaration of other safety data ... 36
21.3.3 Annual safety report ... 36
21.4 DATA MONITORING COMMITTEE (DMC) ... 36

22 FINAL STUDY REPORT ... 36

23 ARCHIVING ... 36

24 FUNDING AND INSURANCE ... 37

24.1 FUNDING SOURCES ... 37
24.2 INSURANCE ... 37

25 BIBLIOGRAPHY ... 37

26 LIST OF ADDENDA ... 40

26.1 LIST OF INVESTIGATORS ... 40
26.2 BLOOD SAMPLING AND CONSERVATION ... 41
26.3 EDIT-B PROCESS ... 41

1 GLOSSARY

AUC-ROC	Area under the ROC curve
BD	Bipolar disorder
CPSP	Clinical performance study protocol
CRO	Clinical research organisation
DSM-5	Diagnostic and statistical manual of mental disorders, 5th edition
GCP	Good clinical practice
MADRS	Montgomery-Asberg Depression Rating Scale
MDD	Major depressive disorder (equivalent: unipolar disorder)
MDE	Major depressive episode
MINI	Mini-International Neuropsychiatric Interview
NGS	Next-generation sequencing
RNA	Ribonucleic acid
ROC	Receiver operating characteristics
Se	Sensitivity
Sp	Specificity
YMRS	Young Mania Rating Scale

2 STUDY SUMMARY

Full title	"Clinical validation study for EDIT-B test: an aid for differential diagnosis of bipolar disorder, based on RNA editing blood biomarkers"
Acronym/reference	EDIT-B
Coordinating investigator	Pr Eduard Vieta<br>Hospital Clinic de Barcelona<br>Villarroel, 170<br>08036 Barcelona, Spain<br>Tel: +34-93-227-5477<br>Fax: +34-93-227-9228<br>e-mail: evieta@clinic.cat
Scientific Director	Dinah Weissmann<br>Cap Gamma<br>1682 rue de la Valsière<br>34184 Montpellier, France<br>Tel: +33 4 67 04 74 64<br>E-mail: dweissmann@alcediag-alcen.com
Sponsor	Alcediag
Scientific justification	Differentiation between major depressive disorder (MDD) and bipolar disorder (BD) as soon as possible in the patient journey represents a major clinical issue. When the patient is in a depressive phase, the symptoms are similar between the two pathologies and the current clinical scales fail in distinguishing them. Physicians often report this difficulty and as a consequence, the mean time from onset to bipolar disorder diagnosis is currently 7.5 years. These diagnostic delays and misdiagnosis lead to damaging consequences for patients and their loved ones: worsening of symptoms, comorbidities, suicide risk and inadequate care resulting in severe impairment in social and occupational functioning. Faced with these high expectations for accurate diagnostic methods for an earlier management of psychiatric patients, the combination of relevant clinical features and biomarkers could stand for a solution, leading to a personalised approach in patients with mood disorders.<br>In a first clinical discovery study, a panel of RNA biomarkers in the blood of patients with a major depressive episode (MDE) has been identified, allowing to differentiate bipolar disorder from MDD (unipolar depression). These biomarkers are based on RNA modifications, namely RNA editing, that could be identified using molecular biology, NGS and artificial intelligence. This panel constitutes EDIT-B test, which is

	based on Alcediag's proprietary and patented biomarkers and algorithms.<br><br><u>Preliminary results</u><br>Recently, we identified 646 variants (366 genes) differentially edited between patients with depression and healthy volunteers from a discovery cohort of 57 participants. After using stringent criteria and biological pathway analysis, a panel of biomarker candidates were singled out and tested in a validation cohort of 410 participants.<br>In a second step, by using this panel of RNA edited blood and testing a cohort of 255 patients with depression including 95 BD patients in a depressive episode (with precise criteria of depression), we identified a combination of biomarkers which allowed a differential diagnosis of bipolar disorder with an area under the ROC curve (AUC-ROC) of 0.899 and high specificity (Sp=83.7%) and sensitivity (Se=84.8%). In a further step, we selected three different signatures showing high performance. The association of RNA editing variants modifications with depression subtypes and the use of artificial intelligence allowed developing a new tool to identify, among patients with depression, those suffering from BD. This test will help to reduce the misdiagnosis delay of bipolar patients, leading to an earlier implementation of a proper treatment [1].
Objective and endpoint	<u>Objective</u>: The objective of this study is to identify among three signatures the best one in terms of external validation of the diagnostic performances (sensitivity, specificity and AUC-ROC) on MDD (unipolar) versus bipolar disorder differentiation in patients with depression.<br><br><u>Endpoint</u>: Categorisation of the patient according to the signature as patient with MDD (unipolar) or bipolar disorder
Design of the study	The study is a cross-sectional design which includes patients with depression suffering from MDD (unipolar) or bipolar depressive disorder.<br>Ratio of the two populations is aimed for 1:1, but accepted up to 2:1 reflecting the imbalance in real-life conditions.
Population of study participants	Patients with depression meeting the major depressive episode (MDE) criteria listed in Diagnostic and Statistical

	Manual of Mental disorders 5 (DSM-5) using the Mini-International Neuropsychiatric Interview (MINI) [2].
Inclusion criteria	1. Signed informed consent.<br>2. Male or female ≥ 18 and ≤ 80 years of age at inclusion.<br>3. Montgomery-Asberg Depression Rating Scale (MADRS) ≥ 20<br>4. In- and out-patients can be recruited.<br>5. Diagnosed with MDE with the MINI for DSM-5<br>6. Currently treated for the MDE<br>7. Diagnosed with MDD (unipolar) or bipolar disorder with the MINI for DSM-5.<br>8. For patients with BD: at least one manic or hypomanic episode<br>9. For patients with MDD (unipolar): at least one MDE
Exclusion criteria	1. MDD (unipolar) patients with first degree family history of bipolar disorder<br>2. Young Mania Rating Scale (YMRS) > 12<br>3. Pregnant women<br>4. Unipolar or bipolar depression secondary to major central nervous system affections, including infections in the brain, tumours of the brain, stroke, Alzheimer's disease, Parkinson's disease, Multiple sclerosis, other major brain affections<br>5. Schizo-affective patients
Device(s) under investigation	Name of the test: EDIT-B<br><br><u>Intended use</u><br>EDIT-B is a blood test system which assists the physicians in the process of differentiating bipolar disorder from major depression disorder.<br><br><u>Description of the test</u><br>EDIT-B combines sequencing data related to RNA editing on biomarkers, and individual data (age, sex, treatment, tobacco use and alcohol abuse), to determine a score to aid in the differential diagnosis of MDD (unipolar) and bipolar disorder.<br>From a blood sample collected at usual collection points, the biological analyses are carried out by laboratories accredited for RNA sequencing and approved by the company ALCEDIAG.<br><br>The test EDIT-B consists of the kit EDIT-B, the appropriate protocol for biological processing and an

	access to the EDIT-B platform to upload sequencing data and to receive the test result that is generated with ALCEDIAG software.
Comparator arm	NA
Additional interventions added for the study	- Mini-International Neuropsychiatric Interview (MINI) (to conclude/validate the objective) [2]<br>- Montgomery-Asberg Depression Rating Scale (MADRS) [3]<br>- Young Mania Rating Scale (YMRS) [4]<br>- EQ-5D-5L quality of life questionnaire [5]<br>- Two blood samples of 2.5 ml each will be collected in PAXgene® tubes (Specific procedures)
Expected benefits for the participants and for society	Patients benefit from a throughout diagnostic evaluation including a MINI interview by participating in the study.<br>From a society perspective, the diagnostic tools based on the signatures composed of RNA editing-based biomarkers patented by Alcediag could improve patient diagnosis in the context of mood disorders, including MDD (unipolar) and bipolar disorder. Given the urgent need for accurate diagnostic methods for an earlier management of psychiatric patients, this study would contribute to the development of innovative solutions, leading to a personalized approach in patients with mood disorders.
Risks and burdens added by the study	Burden added by the study is only the collection of two additional 2.5 ml blood samples during routine blood sampling.<br>Risk level of the study is (A).
Practical implementation	
Number of participants included	**436 patients with depression:**<br>- 218 patients with diagnosed MDD (unipolar)<br>- 218 patients with diagnosed bipolar disorder
Number of centres	This a multicentre international study.<br>4 Expert clinical centres:<br>- Hospital Clinic de Barcelona, Spain<br>Pr Eduard Vieta, Principal Investigator<br>- Capital Region of Denmark, Denmark<br>Pr Lars Kessing, Investigator<br>- GHU Paris Psychiatrie & Neurosciences, France<br>Pr Chantal Henry, Investigator<br>- Parc Sanitari Sant Joan de Déu, Spain<br>Pr Josep Maria Haro, Investigator

Duration of the study	- inclusion period: 32 months<br>- participation period 1 day (duration of the medical consultation)<br>- total duration 32 months + 1 day
Number of enrolments expected per site and per month	3.5 patients
Statistical analysis	The objective of this study is to estimate three EDIT-B signatures in term of their external validity. For this purpose, performances of the tests will be estimated by calculating for each signature its Sensitivity (i.e. the probability that a test will indicate 'bipolar disorders' among those with this disease), Specificity (i.e. the fraction of those without bipolar disorders who will have a negative test result) and its Accuracy to predict the diagnosis of bipolar disorders. In addition, Area under the ROC curves of each EDIT-B signature will be calculated.<br>The categorisation of the type of disorder (MDD vs. BD) by experts will be based on the MINI considered as the gold standard for diagnosis.<br>The performances of the tested signatures will be compared on a two-by-two basis by comparing sensitivities, specificities and accuracy, using the methods based on Mac Nemar test [6]. AUC-ROC of the three signatures will also be compared by DeLong method [7].<br>In addition, for exploratory purposes, multivariate logistics models will be used to identify patients' characteristics that can potentially affect the concordance between the classification proposed by the signature and those of the experts.<br><br>We calculated that a total sample size of N=392 patients with depression with a 1:1 ratio of MDD (unipolar) vs. bipolar disorder. This will allow to estimate the four parameters of performances with a precision of ± 5% or better (considering that the value of specificity is the lowest of the four expected values of performance parameters, precision of the estimates will be better for these three latter). We used the methods proposed by Buderer and Jones for these calculations [8].<br>Considering a maximal 10% attrition rate, N=436 patients will be included.
Funding sources	EIT (European institute of Innovation & Technology) Health, clinical centres, Synlab and Alcediag.

Study will have a Data Monitoring Committee	Given that the patients' diagnosis has been determined according to the highest applicable clinical standards, before the implementation of the in vitro diagnostic medical device under investigation, the foreseeable risk incurred through participating in the research is negligible. Therefore, the constitution of a Data Monitoring Committee (DMC) is not envisaged for the study.
Normative references	**This CPSP is designed according to the requirements of the ISO 20916:2019 standard and GCP**

This CPSP is designed according to the requirements of the ISO 20916:2019 standard.

3 SCIENTIFIC JUSTIFICATION FOR THE STUDY

3.1 Hypothesis for the study

In a first clinical discovery study, a panel of RNA biomarkers in the blood of patients with a major depressive episode (MDE) has been identified, allowing to differentiate bipolar disorder (BD) from major depressive disorder (MDD, unipolar depression). These biomarkers are based on ribonucleic acid (RNA) modifications, namely RNA editing, that could be identified using molecular biology, next-generation sequencing (NGS) and artificial intelligence. This panel constitutes EDIT-B test, which is based on Alcediag's proprietary and patented biomarkers and algorithms.

The present study aims to validate the biomarker signatures proposed by Alcediag by measuring the association between the modifications of the RNA editing and unipolar/bipolar diagnosis, in patients with a MDE in real-life setting pilot centres.

3.2 Description of knowledge relating to the condition in question

Differentiation between MDD (unipolar) and BD as soon as possible in the patient journey represents a major clinical issue. When the patient is in a major depressive episode, the symptoms are similar between the two pathologies and the current clinical scales fail in distinguishing them. Physicians often report this difficulty and as a consequence the mean time from onset to BD diagnosis is currently 7.5 years. These diagnostic delays and misdiagnosis lead to damaging consequences for patients and their loved ones: worsening of symptoms, comorbidities, suicide risk and inadequate care resulting in severe impairment in social and occupational functioning. Faced with these high expectations for accurate diagnostic methods for an earlier management of psychiatric patients, the combination of relevant clinical features and biomarkers could stand for a solution, leading to a personalised approach in patients with mood disorders.

3.3 Summary of relevant pre-clinical experiments and clinical trials

Bipolar disorder is characterised by alternating mood states, from depressive episodes to hypomanic or manic episodes [9-11]. BD is associated with functional impairment, high disability, premature mortality due to medical comorbidities, diabetes, metabolic syndrome or cardiovascular disease [12-14]. Importantly, a pro-inflammatory status underlies the high comorbidity of BD. Converging studies have highlighted the role of peripheral immune activation in the pathophysiology of the disease [15, 16]. One of the most important key mechanisms involved in the regulation of innate immune system and thus in inflammatory response is the RNA editing mediated by ADAR1 (adenosine deaminase acting on RNA) [17, 18]. RNA editing makes the link between peripheral inflammation and the central nervous system (CNS), allowing to diversify the transcriptome. The most conserved editing events occur in transcripts encoding synapse-associated proteins. Introducing codon changes in mRNAs encoding key proteins for nervous system activity, including glutamate and serotonin receptors, has been shown to modulate their activity [19, 20]. The A-to-I editing of serotonin

receptor subtype 2C (5-HTR2c) mRNA leads to the dominant expression of the 5-HTR2c isoform which has reduced G-protein coupling functions and may be causative in the pathophysiology of depression [21]. Recently, Alcediag has identified in the anterior cingulate cortex (Brodmann Area 24) of depressed suicide decedents modifications of editing in mRNA of 5-HTR2c [22] and PDE8A (PhosphoDiEsterase 8A) [23]. Interestingly, phosphodiesterases, a key modulator of signal transduction downstream 5-HTR2c, are involved in inflammatory cell activation, memory and cognition [24]. More recently, Alcediag demonstrated the diagnostic value of PDE8A in depression, in the blood from hepatitis C virus patients treated with interferon [25], and from patients with depression and suicide attempters compared to age-matched and sex-matched healthy controls [26].
Based on these results that demonstrate the value of RNA editing modifications for diagnostic purposes, we identified 646 variants (366 genes) differentially edited between patients with depression and healthy volunteers from a discovery cohort of 57 participants. After using stringent criteria and biological pathway analysis, a panel of biomarker candidates was singled out and tested in a validation cohort of 410 participants. Combining the selected biomarkers with a machine learning approach achieved to discriminate patients with depression versus controls with an area under a receiver operating characteristic (ROC) curve (AUC-ROC) of 0.930 (confidence interval (CI) 95% [0.879-0.982]), a sensitivity (Se) of 84.0% and a specificity (Sp) of 87.1%.
In a second step, by using this panel of RNA edited blood and testing a cohort of 255 patients with depression including 95 BD patients in a major depressive episode (with precise criteria of depression), we identified a combination of biomarkers which allowed a differential diagnosis of bipolar disorder with an of 0.899 and high specificity (Sp=83.7%) and sensitivity (Se=84.8%).
In a further step, we selected three different signatures showing high performance.

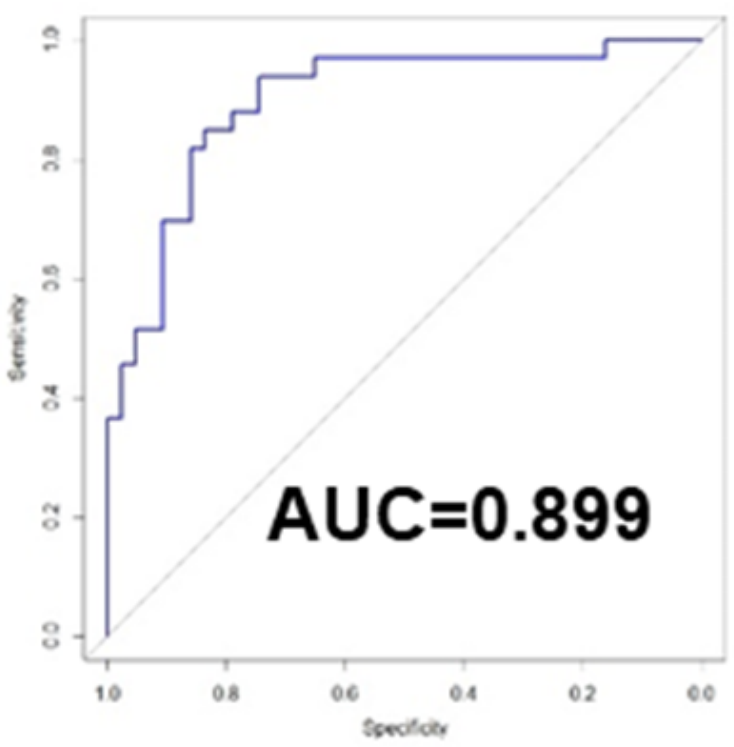

	Performance
AUC	**0.899**
CI 95%	[0.826 ; 0.971]
Se	**84.8%**
Sp	**83.7%**
PPV	80.0%
NPV	87.8%
Acc	84.2%
Cutoff	0.5

Figure 1: AUC-ROC of one of the selected signatures. (Salvetat et al., Molecular Psychiatry, Submitted).

The association of RNA editing variants modifications with depression subtypes and the use of artificial intelligence allowed developing a new tool to identify, among patients with depression, those suffering from BD. This test, EDIT-B, will help to reduce the misdiagnosis delay of bipolar patients, leading to an earlier implementation of a proper treatment [1].

4 IVD MEDICAL DEVICE UNDER INVESTIGATION

4.1 Summary description of the IVD medical device under investigation

The test EDIT-B consists of the kit EDIT-B, the appropriate protocol for biological processing and an access to the EDIT-B platform to upload sequencing data and to receive the test result that is generated with ALCEDIAG software.

4.2 Identification and instructions for use

4.2.1 Name of the IVD medical device

Name of the test: EDIT-B
The test EDIT-B consists of the kit EDIT-B, the appropriate protocol for biological processing and an access to the EDIT-B platform to upload sequencing data and to receive the test result that is generated with ALCEDIAG software.

4.2.2 Intended purpose of the device

EDIT-B is a blood test system that assists the physicians in the process of differentiating bipolar disorder from major depression disorder.

A blood sample of two times 2.5 ml drawn using PAXgene® Blood RNA tubes (Becton Dickinson, UK) is used for testing.

EDIT-B is intended to be used on patients aged from 18 to 80 years old, male or female, with a major depressive episode and being treated for that depression at the moment of the test.

Technical aspects regarding the use of the kit concerns only Synlab laboratory and will be added in a specific documentation.

4.3 Required training and experience

At the clinician level the use of the device is limited to a standard blood sampling and does not require any specific skill or learning curve.

5 SPONSOR

ALCEDIAG
Cap Gamma, 1682 rue de la Valsière, 34184 Montpellier, France
Tel: +33 4 67 04 74 64
E-mail: dweissmann@alcediag-alcen.com
www.alcediag-alcen.com

6 STUDY SITES

This is a **multicentre international** study

- Recruitment centres

  Four expert clinical centres:
  Hospital Clinic de Barcelona; Pr Eduard Vieta, Coordinating and Principal Investigator (PI)
  Capital Region of Denmark; Pr Lars Kessing, PI
  GHU Psychiatrie & Neurosciences; Pr Chantal Henry, PI
  Parc Sanitari Sant Joan de Déu; Pr Josep Maria Haro, PI

- Central laboratory

  Synlab Italia – Gebetic Unit; via Beato L. Pavoni 18, 25014 Castenedolo (BS), Italy

6.1 Principal investigator responsibilities

The responsibilities of the principal investigator are defined in accordance with requirements specified in 5.5.2 of ISO 20916:2019 standard. Before starting the study, each investigator will give the sponsor's representative a copy of his/her updated personal curriculum vitae, signed and dated less than one year, with his/her professional number. The CV must include any previous involvement in clinical research and related training.

Each investigator will commit to comply with legislation and to conduct the study in line with GCP, in accordance with the Declaration of Helsinki.

The Principal Investigator at each participating site will sign a commitment of responsibility document which will be sent to the sponsor's representative.
The investigators and their staff will sign a delegation of duties form specifying each person's role and will provide their CVs.

7 OBJECTIVES OF THE CPSP

7.1 Study Objective

The objective of this study is to identify among three signatures the best one in terms of external validation of the diagnostic performances (sensitivity, specificity, accuracy and AUC-ROC) on MDD (unipolar) versus bipolar disorder differentiation in patients with depression.

7.2 Endpoint

Categorisation of the patient according to the signatures as patient with MDD (unipolar) or bipolar disorder.

7.3 Claims and intended performance of the IVD medical device under investigation that are to be evaluated

The claims and intended performance of EDIT-B test are to differentiate bipolar disorder from major depressive disorder. The performances of sensitivity, specificity and AUC-ROC should be close to those observed in the first study, i.e. 90%, 85%, 93% respectively.

7.4 Risks and benefits

Patients benefit from a throughout diagnostic evaluation including a MINI interview by participating in the study. Given that the patients' diagnosis has been determined according to the highest applicable clinical standards before the implementation of the in vitro diagnostic medical device under investigation, the foreseeable risk incurred through participating in the research is negligible.
Note that from a society perspective, the diagnostic tools based on the signatures composed of RNA editing-based biomarkers patented by Alcediag could improve patient diagnosis in the context of mood disorders, including depression and bipolar disorder. Given the urgent need for accurate diagnostic methods for an earlier management of psychiatric patients, this study would contribute to the development of innovative solutions, leading to a personalised therapeutic approach in patients with mood disorders.

7.5 Description of research methodology

7.5.1 Design of the study

The study is a cross-sectional design which includes patients with depression suffering from MDD (unipolar) or bipolar disorder. Ratio of the two populations is aimed for 1:1. However, in real-life conditions, there are more unipolar than bipolar disorder patients. Thus, a ratio imbalance, reflecting the real-life conditions, is accepted up to 2:1.

7.5.2 Identification of participants

The participants in this research will be identified as follows:
Site number (3 digits) - Sequential enrolment number for the site (3 digits)
This reference number is unique and will be used for the entire duration of the study.

8 IVD COMPARATOR

NA

9 STUDY POPULATION AND SPECIMENS

9.1 Validated specimen type

A blood sample of 2 x 2.5 ml will be drawn using two PAXgene® Blood RNA tubes (Becton Dickinson, UK) according to the manufacturer's instructions.

9.2 Inclusion criteria

1. Signed informed consent.
2. Male or female ≥ 18 and ≤ 80 years of age at inclusion.
3. MADRS ≥ 20
4. In- and out-patients can be recruited.
5. Diagnosed with MDE with the MINI for DSM-5
6. Currently treated for the MDE
7. Diagnosed with MDD (unipolar) or bipolar disorder with the MINI for DSM-5.
8. For patients with bipolar disorder: at least one manic or hypomanic episode
9. For patients with MDD (unipolar): at least one MDE

9.3 Exclusion criteria

1. MDD (unipolar) patients with first degree family history of bipolar disorder
2. YMRS > 12
3. Pregnant women
4. Unipolar or bipolar depression secondary to major central nervous system affections, including infections in the brain, tumours of the brain, stroke, Alzheimer’s disease, Parkinson’s disease, Multiple sclerosis, other major brain affections
5. Schizo-affective patients

9.4 Information necessary to characterise the subject

To ascertain the robustness of the diagnosis and to avoid mixed episodes, the following patients will be included: Patients with depression meeting the MDE criteria listed in Diagnostic and Statistical Manual of Mental disorders 5 (DSM-5) using the Mini-International Neuropsychiatric Interview (MINI) [2] and with Montgomery-Asberg Depression Rating Scale (MADRS) ≥ 20. For patients with BD at least one manic or hypomanic episode is required, for patients with MDD (unipolar) at least one MDE is required.

9.5 Number of subjects

Patients with depression meeting the MDE criteria in DSM-5 using the MINI will be eligible for the study. In each centre, MINI training at hospitals for participating investigators will be performed prior the beginning of the study.

Total number of participants to be included	436
Number of centres	4
Enrolment period (months)	32
Number of participants/centres	109*
**Number of participants/centre/months**	**3.5***

*Enrolment is competitive and centres are not limited in enrolment numbers. Thus, 109 and 3.5 are approximative values assuming a balanced enrolment throughout the centers.

9.6 Specimen storage, handling, transport, and disposal

9.6.1 Blood samples

The PAXgene® Blood RNA tubes with collected blood samples will be stored upright at room temperature (18−25˚C) for 2 hours before transferring to freezer (−20˚C). The samples will be stored locally at -20°C and sent on dry ice every two months to the Genetic Unit of Synlab Italia laboratory (via Beato L. Pavoni 18, 25014 Castenedolo (BS), Italy) using time- and temperature-controlled transport. After reception, blood samples should be kept -80°C at Synlab laboratory upon subsequent analysis.

One of the two collected blood samples of the first 30 patients will be sent from Synlab Italia to Veracyte (163 avenue de Luminy, 13009 Marseille, France), for finalizing the analytical performance of EDIT-B kit, an evaluation that requires patient samples.

Veracyte is contractually bound to ALCEDIAG, and is submitted to GDPR clauses. This third party is following the GDPR regulation and improves all security and technical measures according to the regulation.

The blood samples will only be used for the purpose of the study. Once the analysis is finished all of these remaining blood samples and any other sample will be directly destroyed by Veracyte.

9.6.2 Biological samples collection

RNA samples extracted from the blood taken as part of the study will be stored in a biological sample collection.Analyses will be performed per batch of 100 patients.

During the study, the sample collection will be stored at the Genetic Unit of Synlab Italia laboratory (via Beato L. Pavoni 18, 25014 Castenedolo (BS), Italy) under the supervision of Christina Lapucci for a period of five years. Technical aspects regarding the use of the kit concerns only Synlab laboratory and will be added in a specific documentation.

At the end of the study, extracted RNA will be kept for a period of five years and may be used for additional studies if patients agree with that point in consent form.
At the end of the study, the samples may be used for further analysis, not described in the initial protocol but which may be useful for our investigation in light of advances in scientific knowledge, provided the participant is informed and does not oppose this use, as stated in the information note/consent form.

**Type of sample**	**Quantity**	**Storage location** (name and entity)	**Supervisor of the sample collection** (name and entity)	**Purpose of the sample collection**	**Storage duration**	**End use/Future (destruction, etc.)**
Blood (RNA)	2x2.5 ml	Synlab Italia – Genetic Unit; via Beato L. Pavoni 18, 25014 Castenedolo (BS), Italy	Christina Lapucci Synlab	Research: Validation of the diagnostic power of the algorithm on unipolar or bipolar differentia-tion in patients with depression	Five years	Destruction or use for additional studies (depending on patient decision in consent form)

10 IMPLEMENTATION OF THE STUDY

The study will be conducted in compliance with Good Clinical Practices, the principles of which are addressed in the ICH Guideline for good clinical practice E6(R2).
According to current national legal and statutory provisions, before any examination or intervention related to the study may be carried out, the investigator must obtain the freely given, informed and written consent of the participant.

The patient participation will consist in a single visit that will last the time of medical consultation during which the samples required for the assay by medical device under investigation will be collected.

10.1 Inclusion and last study visit

The patient recruitment will be realised during a visit scheduled as part of the standard of care.

Before any intervention, test or procedure specific to the study, participants will be informed of the objectives and the additional examinations specific to the study. The content of the information provided to the patient will comply with the requirements presented in 5.8.4 of ISO 14155:2020 standard.

Whose consent must be obtained	Who informs the individuals and collects their consent	At what point the individuals are informed	At what point the consent is obtained
• the individual participating in the study	• the principal investigator or collaborating physician (psychiatrist), declared and trained in the study)	• standard of care visit	• after the patient has signed the consent form

The inclusion and non-inclusion criteria will be verified, and the patients will be asked to sign the consent.

Following clinical parameters will be collected:

- sociodemographic data
    - Note that the test EDIT-B is based on the measurement of RNA editing, that is an enzymatic mechanism catalysed by specific enzymes, so-called ADAR (Adenosine deaminases acting on RNA). It has been shown that this enzyme activity could be modulated according to the ethnicity of the patients [27]. For this reason, ethnical information will also be collected.
- characteristics of disease
  characteristics of current episode
  current treatment
  comorbidity
  family psychiatric history
  toxic consumption
- Montgomery-Asberg Depression Rating Scale (MADRS) score [3]
- Young Mania Rating Scale (YMRS) score [4]
- MDD (unipolar) or bipolar disorder will be assessed by clinician's expertise based on history of the disease and by Mini-International Neuropsychiatric Interview (MINI) that will be a gold standard for diagnosis [2]
- EQ-5D-5L will be used as quality of life questionnaire to evaluate current quality of life and retrospectively, quality of life before correct diagnosis [5]

Blood sample of an additional volume of 2 x 2.5 ml will be collected during routine blood sampling at the time of the visit.

10.2 Duration of the study.

Duration of enrolment period	32 months
The length of participation for participants	1 day
Total study duration:	32 months + 1 day

10.3 Distinction between standard care and study

TABLE: "Standard care" vs. "additional interventions" required specifically for the study

Interventions, procedures and treatments carried out for research purposes	Interventions, procedures and treatments associated with standard care	Interventions, procedures and treatments added for research purposes
Visits	Inclusion visit	
MINI interview		At the inclusion visit
Montgomery-Asberg Depression Rating Scale (MADRS)		At the inclusion visit
Young Mania Rating Scale (YMRS)		At the inclusion visit
EQ-5D-5L questionnaire		At the inclusion visit
Blood samples		2 additional tubes of 2.5 ml each (5 ml in total)

10.4 Termination rules

10.4.1 Criteria and procedure for premature withdrawal of a participant from the study

- Note that for each patient study duration is limited to one day (i.e. no follow-up).
- Participants may exit the study at any time and for any reason.
- The investigator can temporarily or permanently withdraw a participant from the study for any reason or if it is in the participant’s best interests.

10.4.2 Follow-up of participants following premature withdrawal from the study

NA

10.4.3 Procedures for replacing participants, if applicable

Participants for whom blood sample will not be available will be replaced.

10.4.4 Full or partial discontinuation of the study

Sponsor or the Competent Authority (from each participating country) can prematurely discontinue all or part of the study, temporarily or permanently.

Similarly, sponsor or the Competent Authority (from each participating country) may decide to prematurely discontinue the study due to new information about the intervention performed or the product, in light of which the objectives of the study or clinical programme are unlikely to be achieved.

Sponsor reserves the right to permanently suspend enrolment at any time if it appears that the inclusion objectives are not met.

11 STUDY PROCEDURES

11.1 Sample collection and processing

A blood sample of two times 2.5 ml will be drawn using PAXgene® Blood RNA tubes (Becton Dickinson, UK) according to the manufacturer's instructions.
Technical aspects regarding the use of the kit concerns only Synlab laboratory and will be added in a specific documentation.

In the present study all the clinical and biological data will be collected during a unique visit.

For practical reason, blood samples will be sent to Synlab every two months by lot of 18 tubes per centre, corresponding to nine patients (two tubes per patient). Thus, Synlab will receive and store at -80°C 72 tubes per month (18 tubes per clinical centre, four clinical centres).

One of the two collected blood samples of the first 30 patients will be sent from Synlab to Veracyte, contractually bound to Alcediag and following GDPR regulation, for finalizing the analytical performance of EDIT-B kit, an evaluation that requires patient samples. The blood samples will only be used for the purpose of the study. Once the analysis is finished all of these remaining blood samples and any other sample will be directly destroyed by Veracyte.

Methods for blood samples conservation and their processing to evaluate RNA editing modifications of different targets and use of the three versions of algorithm are described in appendix 26.2 and 26.3.

All the biological processing of the blood samples and use of algorithm allowing the determination of the categorisation will be made without any knowledge of the experts' diagnosis.

In addition, both global and individual results of the signatures will not be communicated to the investigators before the end of the study.

11.2 Procedures associated with use of the device

EDIT-B combines sequencing data related to RNA editing on biomarkers, and individual data (age, sex, treatment, tobacco use and alcohol abuse), to determine a score to aid in the differential diagnosis of MDD (unipolar) from bipolar disorder.

From a blood sample collected at usual collection points, the biological analyses are carried out by laboratories accredited for RNA sequencing and approved by the company Alcediag. Then the score calculation is carried out by a software developed by Alcediag, which contains modules for quality control and an algorithm to determine the patient's score, based on a mathematical proprietary model. The score calculation algorithm is hosted on a SaaS (Software as a Service) type platform, accessible via the website http://edit-b.alcediag-alcen.com/. The platform offers dedicated access to medical biology laboratories (access authorisation administered by the company Alcediag). The platform complies with European security and health data management standards.

11.3 Description of performance or efficacy assessment parameters

Performances of the tests will be estimated by calculating their sensitivity (i.e. the probability that a test will indicate 'bipolar disorders' among those with this disease) and specificity (i.e. the fraction of those without bipolar disorders who will have a negative test result). In addition, area under the ROC curves (AUC-ROC) of the signatures will be calculated.

12 <u>MONITORING PLAN</u>

12.1 Quality assurance

12.1.1 General organisation

The sponsor must ensure the safety and respect of individuals who have agreed to participate in the study. The sponsor will implement a quality assurance system to best monitor the implementation of the study in the investigation centres.

For this purpose, the sponsor shall assign Clinical Research Organization (CRO) whose primary role is to carry out regular follow-up visits at the study locations, after having carried out the initial visits.
The purpose of monitoring the study, as defined in Good Clinical Practices (GCP section 5.18.1), is to verify that:

- the rights, safety and protection of the research participants are met
- the data reported are exact, complete and consistent with the source documents
- the study is carried out in accordance with the protocol in force, the GCPs and the statutory and regulatory provisions in force.

12.1.2 Strategy for centre opening

All participating centres will be opened within a two months period after authorisations of the study by competent authority and EC associated with the centre.

12.1.3 Scope of centre monitoring

The sponsor shall determine the extent and nature of monitoring appropriate for the clinical investigation based on the risk assessment. The extent and nature of the monitoring, including the strategy for source data verification versus centralized data review (evaluation without visiting the investigation site), subject protection and timely reporting, shall be based on the

objective, design, complexity, size, critical data points and endpoints of the clinical investigation and the degree of deviation from normal clinical practice – risk-based monitoring.

Therefore, in the case of this A risk study, in agreement with the coordinating investigator, the sponsor has determined the logistical score and impact, resulting in a study monitoring level to be implemented: minimum level.

12.1.4 Quality control

AIXIAL a Clinical Research Organisation (CRO) appointed by the sponsor will be responsible for the good completion of the study, for collecting, documenting, recording and reporting all handwritten data, in accordance with Good Clinical Practices as well as the statutory and regulatory requirements.

The investigator and the members of the investigator's team agree to make themselves available during regular Quality Control visits carried out by the Clinical Research Associate.
During these visits, the following elements will be reviewed depending on the monitoring level:

- written consent
- compliance with the study protocol and with the procedures defined therein
- quality of the data collected in the case report form: accuracy, missing data, consistency of the data with the "source" documents (medical files, appointment books, original copies of laboratory results, etc.)
- management of the devices used

12.1.5 Case report forms

All information required by the protocol must be entered in the case report forms. The data must be collected as and when they are obtained, and clearly recorded in these case report forms. Any missing data must be coded.
Every site will have access to the electronic case report forms via a web-based data collection system. Investigators will be given a document offering guidance on using this tool.
When the investigators complete the case report form via the Internet, the CRA can view the data quickly and remotely. The investigator is responsible for the accuracy, quality and relevance of all the data entered. In addition, the data are immediately verified as they are entered, thanks to consistency checks. To this end, the investigator must validate any changes to the values in the case report form. An audit trail will be kept of all changes. A justification can be added when applicable, as a comment.
A print-out, authenticated (signed and dated) by the investigator, will be requested at the end of the study. The investigator must archive a copy of the authenticated document that was issued to the sponsor.

12.2 Audits and inspections

The investigators agree to consent to the quality assurance audits carried out by the sponsor as well as the inspections carried out by the competent authorities. All data, documents and reports may be subject to regulatory audits and inspections. These audits and inspections cannot be refused on the grounds of medical secrecy.

An audit can be carried out at any time by individuals appointed by the sponsor and independent of those responsible for the research. The audits will be conducted in accordance with the recommendations provided in Annex I of the ISO 20916:2019 standard. The aim of

the audits is to ensure the quality of the study, the validity of the results and compliance with the legislation and regulations in force.

The people who manage and monitor the study agree to comply with the sponsor's requirements and with the competent authority regarding study audits or inspections.

The audit may encompass all stages of the study, from the development of the protocol to the publication of the results, including the storage of the data used or produced as part of the study.

13 SPECIFIC STUDY COMMITTEES

13.1 Steering Committee

- Committee members:
  - Pr Eduard Vieta, Hospital Clinic de Barcelona
  - Pr Lars Kessing, Capital Region of Denmark
  - Pr Chantal Henry, GHU Psychiatrie & Neurosciences
  - Pr Josep Maria Haro, Parc Sanitari Sant Joan de Déu
  - Sponsor representative: Dinah Weissmann; Jean-Daniel Abraham
- Roles: the overall structure of the study, coordinate information, determine the initial methodology and oversee the study
- Operating procedures: meetings at a place adapted to the protocol and the occurrence of events (at least one every six months).

14 DATA MANAGEMENT

14.1 Data collection procedures

Demographic and clinical information will be collected in the e-CRF designed using the electronic data capture (EDC) means provided by AIXIAL (i.e. the sponsor's CRO designee), according to requirements given in Annexe A5 of ISO 20916:2019 standard.
It is the responsibility of the investigator to maintain adequate and accurate e-CRF designed by the Sponsor/ authorized designee to record all observations and other data pertinent to the clinical investigation in a timely manner.
Should a correction be made, the corrected information will be entered in the e-CRF overwriting the initial information. An audit trail will allow identifying the modification.
Data will be available within the system to the Sponsor/ authorized designee as soon as they will be entered in the e-CRF.
The computerized handling of the data by the Sponsor/ authorized designee may generate additional requests to which the investigator will be obliged to respond by confirming or modifying the data questioned. The requests with their responses will be managed through the e-CRF.

AIXIAL will provide access to the EDC system for the duration of the trial through a password-protected method of internet access. Such access will be removed from investigator sites at the end of the site's participation in the study. Data from the EDC system will be archived on appropriate data media (CD-ROM, USB key) and provided to the investigator at that time as a

durable record of the site's e-CRF data. It will be possible for the investigator to make paper printouts from that media.

14.2 Identification of data recorded directly in the CRFs which will be considered as source data

NA

14.3 Right to access data and source documents

14.3.1 Data access

In accordance with GCPs:
- the sponsor is responsible for ensuring all parties involved in the study agree to guarantee direct access to all locations where the study will be carried out the source data, the source documents and the reports, for the purposes of the sponsor's quality control and audit procedures or inspections by the competent authority
- the investigators will ensure the persons in charge of monitoring, quality control and auditing or inspecting the study involving human participants have access to the documents and personal data strictly necessary for these tasks, in accordance with the statutory and regulatory provisions in force.

14.3.2 Source documents

Source document is defined as original or certified copy of printed, optical or electronic document containing source data. These documents will be kept in accordance with the regulations in force by the investigator or by the hospital in the case of a hospital medical file.

Source data are defined as all information in original records, certified copies of original records of clinical findings, observations, or other activities in a clinical investigation, necessary for the reconstruction and evaluation of the clinical investigation. Source documents are those in which patient data are recorded and documented for the first time. They include, but are not limited to, hospital records, clinical and office charts, laboratory notes, memoranda, patient-reported outcomes, evaluation checklists, pharmacy dispensing records, recorded data from automated instruments, copies of transcriptions that are certified after verification as being accurate and complete, microfiche, photographic negatives, microfilm or magnetic media, X rays, patient files, and records kept at pharmacies, laboratories, and medico technical departments involved in a clinical trial. Data collected during this study must be recorded on the appropriate source documents.

To facilitate source data verification, the investigators and institutions must provide the Sponsor direct access to applicable source documents and reports for trial related monitoring, Sponsor audits, and IRB/EC review. The study site must also allow inspection by applicable health authorities. The ICF will include a statement by which the patient allows to have direct access to original medical records. These personnel, bound by professional secrecy, must maintain the confidentiality of all personal identity or personal medical information (according to confidentiality and personal data protection rules).

In the context of the study the source documents are:

- medical file
- EDIT-B database with analysed patient's score stored at Alcediag in excel/text file format.

14.3.3 Data confidentiality

The persons responsible for the quality control of clinical studies (Article L.1121-3 of the *Code de la Santé Publique* [French Public Health Code]) will take all necessary precautions to ensure the confidentiality of information relating to the study, the study participants and in particular their identity and the results obtained.
These persons, as well as the investigators themselves, are bound by professional secrecy, as set out in 7.4 of ISO 20916:2019 standard.
During and after the research involving human participants, all data collected concerning the participants and sent to the sponsor by the investigators (or any other specialised collaborators) will be rendered non-identifying.
Under no circumstances shall the names and addresses of the participants involved be shown. Only the participant's initials will be recorded, accompanied by an encoded number specific to the study indicating the order of enrolment.
The sponsor will ensure that each participant has given written permission for any personal information about him or her which is strictly necessary for the quality control of the study to be accessed.

14.4 Data processing and storage of research documents and data

14.4.1 Identification of the data processing manager and location(s)

The data extractions from e-CRF will be made by the Aixial data managers in SAS format and Excel format.

The statistical analyses of demographic and clinical data will be carried out by the statisticians of Aixial under the responsibility of Aixial using the latest version of SAS software.

The NGS data will be generated at Synlab, transferred on Alcediag servers, analysed and stored at Alcediag in excel/text file format under the responsibility of Alcediag. Alcediag processes are GDPR compliant. The final analyses (comparison of eCRF data vs EDIT-B result) will be done at Alcediag once the EDIT-B database will be frozen.

14.4.2 Data entry

Non-identifying data will be entered electronically via a web browser.

14.4.3 Data ownership

ALCEDIAG

15 <u>STATISTICAL CONSIDERATIONS</u>

15.1 Description of statistical methods to be used including the timetable for the planned interim analyses

The objective of this study is to estimate three EDIT-B signatures in term of their external validity. For this purpose, performances of the tests will be estimated by calculating for each signature its Sensitivity (i.e. the probability that a test will indicate 'bipolar disorders' among those with this disease), Specificity (i.e. the fraction of those without bipolar disorders who will have a negative test result) and its Accuracy to predict the diagnosis of bipolar disorders. In addition, Area under the ROC curves of each EDIT-B® signature will be calculated.

The categorisation of the type of disorder (MDD vs. BD) by experts will be based on the MINI considered as the gold standard for diagnosis.
The performances of the tested signatures will be compared on a two-by-two basis by comparing sensitivities and specificity , using the methods based on Mac Nemar test proposed by Hawass [6]. AUC-ROC of the 3 signatures will also be compared by DeLong method [7].

In addition, for exploratory purposes, multivariate logistics models will be used to identify patients' characteristics that can potentially affect the concordance between the classification proposed by the signature and those of the experts.

15.2 Calculation hypotheses for the number of participants required and the result

The sample size will be fixed in order to provide a sufficient precision in the estimates of the performances of the test in terms of sensitivity, specificity, accuracy and AUC-ROC. We hypothesized that the values of sensitivity, specificity and AUC-ROC will be close to those observed in the first study, i.e. 90%, 85%, 93% respectively.

We calculated that a total sample size of N=392 patients with depression with a 1:1: ratio of MDD (unipolar) vs. bipolar disorder. This will allow to estimate the four parameters of performances with a precision of ± 5% or better (considering the that the value of specificity is the lowest of the four expected values of performances parameters, precision of the estimates will be better for these three latter). We used the methods proposed by Buderer and Jones for these calculations [8, 28].

Considering a maximal 10% attrition rate 436 patients will be included.

15.3 Anticipated level of statistical significance

All confidence intervals will be two-sided 95%, and statistical tests comparing performances will be two-sided at 1.66% levels considering a Bonferonni adjustment for multiplicity considering three two-by-two comparisons.

15.4 Statistical criteria for termination of the study.

NA

15.5 Method for taking into account missing, unused or invalid data

Considering the nature of the study all calculation will be made on the valid data without missing value imputation. However, the rate of patients for which the test has been unfeasible will be calculated since representing also an additional important parameter of the test performances in real life conditions.

15.6 Management of modifications made to the analysis plan for the initial strategy.

Any changes to the analysis plan will be described in the Statistical Analysis Plan (SAP) whose last version should be validated before any analysis of the data base.

15.7 Choice of individuals to be included in the analyses

All included patients with a signed inform consent and who did not withdrew it will be analysed.

16 AMENDMENTS TO THE CPSP

Any substantial modification to the protocol by the coordinating investigator must be sent to the sponsor for approval. After approval is given, the sponsor must obtain approval from the EC (Research Ethics Committee) and authorisation from the competent authority within the scope of their respective authorities, before the amendment can be implemented.
The information note and the consent form can be revised if necessary, in particular in case of a substantial amendment to the study or if adverse reactions occur.

17 DEVIATIONS FROM CLINICAL PERFORMANCE STUDY PROTOCOL

The investigator is not allowed to deviate from the CPSP, except when a deviation is necessary to protect subject’s rights, safety and well-being, or the scientific integrity of the clinical performance study. The principal investigator of each participating centre will sign a document that certifies that he or she is committed to comply with the protocol as well as any changes notified to him/her by the sponsor.

Any events that occur as a result of non-compliance – by the investigator or any other individual involved in running the study – with the protocol, standard operating procedures, good clinical practices or statutory and regulatory requirements must be recorded in a declaration of non-compliance and sent to the sponsor.

17.1 Definitions

Minor deviation: dysfunction not likely to affect the organizational requirements of the sponsor and having an impact on the activity itself OR conditions, practices or processes not likely to affect the rights, safety or well-being of subjects involved, or data quality and integrity.

Major deviation: dysfunction that may affect the organizational requirements of the sponsor and having an impact on the activity itself OR conditions, practices or processes that may affect the rights, safety or well-being of the subjects involved in the research or data quality and integrity.

Critical deviation: dysfunction undermining the organizational requirements of the sponsor OR conditions, practices or processes undermining the rights, safety or well-being of the subjects involved in the research or the quality and integrity of the data, and liable to administrative

consequences and criminal prosecution in the event of an inspection. Critical deviations are considered unacceptable.

These non-conformities will be managed in accordance with the promoter's procedures.

17.2 Deviation detection

Any personnel involved in the conduct of the clinical performance study must identify any dysfunction that does not comply with the protocol, collect all tangible elements and the documentation associated with this dysfunction, implement corrections, if necessary.

17.3 Deviation analysis and choice of actions

Any staff involved in the conduct of the clinical performance study must analyse the deviation observed on the following points:

- qualification: minor, major or critical;
- causes;
- the potential or actual impacts, in particular on the security and rights of the subjects and on the quality of the data;
- the solutions to be implemented;
- the choices of corrective actions, aimed at eliminating the cause of a deviation or other undesirable situation detected, and preventive, aimed at eliminating the cause of a potential deviation or other undesirable potential situation.

This analysis is then sent to the head of the division concerned, who validates the proposed analysis, supplemented, if necessary, with the necessary actions or corrections.

17.4 Reporting of major or critical deviations

Any personnel involved in the conduct of the clinical performance study declares major or critical deviation in real time. The declaration deadline is ten days for a major deviation and four days for a critical deviation.
The head of the division analyses the deviation in the event of a recurrence, and takes the appropriate decisions, or even requests an audit or a compliance visit. Treatment of deviation is done within ten days maximum.
The Quality department, in the event of a major or critical deviation, checks the adequacy of the treatment with the sponsor procedures, issues a Quality opinion on the actions carried out, may request additional actions to comply with the sponsor procedures, closes the overall processing of the deviation.
When there are many deviations or several critical deviations, the sponsor assesses the significance and extent of the possible impact. The investigator may be disqualified if he or she has committed multiple critical deviations or has repeatedly deliberately failed to comply with CPSP and / or regulatory requirements.

18 ACCOUNTABILITY OF IVDMD UNDER INVESTIGATION

The principal investigator or an authorised designee shall keep records documenting blood sampling of the patients. These records shall include date and time of blood sampling.

The sponsor shall keep records to document the physical location of all IVD medical devices under investigation, in this case the kit EDIT-B, from shipment of the devices to the central laboratory that processes the blood samples.

The central laboratory shall keep records to document the shipment of the blood samples and for all IVD medical devices under investigation, in this case the kit EDIT-B, the date of receipt, the lot number, the expiry date, the date of use and the date of return to the Sponsor if unused, expired or malfunctioning.

19 STATEMENTS OF CONFORMITY

19.1 Compliance with the Declaration of Helsinki

This clinical performance study shall be conducted in accordance with the ethical principles set out in the World Medical Association's Declaration of Helsinki.

19.2 Request for approval from the Research Ethics Committee

Prior to starting the study, the sponsor shall obtain for this CPSP approval from the appropriate Research Ethics Committee, within the scope of its authority and in accordance with the in force legislation and regulatory requirements.

19.3 Request for authorisation from the competent authority (from each participating country)

Prior to starting the study, the sponsor shall obtain authorisation from regulatory authorities in the country where the clinical investigation is conducted, within the scope of the competent authority and in accordance with the legislation and regulatory requirements.

19.4 Procedures relating to data protection regulations

The computer file used for this research is implemented in accordance with French (amended “Informatique et Libertés” law governing data protection) and European (General Data Protection Regulation – GDPR) regulations.

- Commitment to comply with “Reference Methodology” MR-003 – specific for France

This research is governed by the CNIL (French Data Protection Agency) “Reference Methodology for processing personal data used within the scope of health research” (amended MR-003). The CRO AIXIAL sponsor designee of the research has signed a declaration of compliance with this «Reference Methodology».

20 INFORMED CONSENT PROCESS

According to current national legal and statutory provisions, no clinical research involving human participants can be carried out on a person without his/her freely given and informed consent, obtained in writing after the person has been given the information specified in Annex F4 of ISO 20916:2019 standard.

The individual has the time to reflect after he or she is informed and before signing the consent form.

The person's freely given, written, informed consent will be obtained by the principal investigator or a physician representing the investigator before the person is enrolled in the study.

A copy of the information note and consent form, signed and dated by the research participant and by the principal investigator or the physician representing the investigator will be given to the individual prior to their participation in the study. The principal investigator or the physician representing him/her will keep a copy.

At the end of the study, one copy will be placed in a tamper-proof sealed envelope containing all the consent forms. This envelope will be archived by the sponsor.

In addition, the investigator will specify in the research participant's medical file the methods used for obtaining their consent as well as the methods used for providing information with a view to obtaining their consent. The investigator will retain one copy of the signed and dated consent form.

21 SAFETY ASSESSMENT - RISKS AND BURDEN ADDED BY THE STUDY

21.1 Definitions

According to Article 3 of ISO 20916:2019 standard

- **Adverse event (AE), as defined in 3.2 of ISO 20916:2019 standard**

any untoward medical occurrence, inappropriate patient management decision, unintended disease or injury, or untoward clinical signs in subjects, users, or other persons, with any connection to study related activities, whether or not related to the IVD medical device under investigation.

- **Adverse device effect (ADE), as defined in 3.1 of ISO 20916:2019 standard**

adverse event related to the use of an IVD medical device under investigation.

- **SADE (serious adverse device effect), as defined in 3.43 of ISO 20916:2019 standard**

adverse device effect that has resulted in any of the consequences characteristic of a serious adverse event.

- **SAE (serious adverse event), as defined in 3.44 of ISO 20916:2019 standard**

adverse event that led to any of the following:

a) death,

b) serious deterioration in the health of the subject, users, or other persons as defined by one or more of the following:

1) a life-threatening illness or injury, or

2) a permanent impairment of a body structure or a body function including chronic diseases, or
3) in-patient or prolonged hospitalization, or
4) medical or surgical intervention to prevent life-threatening illness or injury, or permanent impairment to a body structure or a body function,

c) foetal distress, foetal death, a congenital abnormality, or birth defect including physical or mental impairment.

- **device deficiency, as defined in 3.16 of ISO 20916:2019 standard**

inadequacy of a medical device with respect to its identity, quality, durability, reliability, usability, safety or performance.

Note 1 to entry: Device deficiencies include malfunctions, use errors, and inadequacy in the information supplied by the manufacturer including labelling.

Note 2 to entry: This definition includes device deficiencies related to the investigational medical device or the comparator.

21.2 The role of the investigator

During this research, no serious adverse events and defects related to the experimental IVD MD is expected. The adverse events (serious and otherwise) do not need to be reported to the sponsor. The report must instead be made as part of the vigilance procedure applicable to the IVD MD under investigation (medical device vigilance).

Adverse events likely to be related to the treatments prescribed as part of the care during the clinical investigation must be notified by the investigator to the regional pharmacovigilance center on which he depends.

21.2.1 Serious adverse events that require the investigator to notify the sponsor without delay

NA

21.2.2 Specific features of the protocol

NA

21.2.3 Period during which SAEs must be notified without delay by the investigator to the sponsor

NA

21.2.4 Procedures and deadlines for notifying the sponsor

NA

21.3 Role of the sponsor

The sponsor, represented by its Safety Department, shall continuously, throughout the trial, assess the safety of each investigational medical device and/or its procedures for use and/or interventions/procedures/examinations added by the study and any other treatments.

21.3.1 Analysis and declaration of serious adverse events

NA

21.3.2 Analysis and declaration of other safety data

NA

21.3.3 Annual safety report

NA

21.4 Data monitoring committee (DMC)

Given that the patient's diagnosis has been determined according to the highest applicable clinical standards, before the implementation of the IVD MD under investigation, the foreseeable risk incurred through participating in the research is negligible. Therefore, and in accordance with guidance provided in 6.11 of ISO 14155:2020 standard the constitution of a DMC is not considered for the study.

22 FINAL STUDY REPORT

The final report for the research should include the information specified in Annex D of the ISO 14155:2020 standard. The report is written and signed by the sponsor and the investigator. A report summary, drafted according to the reference plan, must be sent to the competent authority within a period of one year following the end of the study, i.e. the end of the participation of the last research participant in the study.

23 ARCHIVING

Specific documents for a clinical study involving human participants concerning an in vitro diagnostic medical device will be archived by the investigator and the sponsor for 15 years after the end of the research.

This indexed archiving includes, in particular:

- A sealed envelope for the investigator containing a copy of all the information notes and consent forms signed by all individuals at the centre who participated in the study;
- A sealed envelope for the sponsor containing a copy of all the information notes and consent forms signed by all individuals at the centre who participated in the study;
- “Study” binders for the investigator and the sponsor, including (non-exhaustive list):

- the successive versions of the protocol (identified by the version number and its date), and any appendices
- the regulatory authorities authorisations and EC (Research Ethics Committee) decisions
- any correspondence
- the enrolment list or register
- the appendices specific to the research
- final study report

➢ The data collection documents

A list of essential clinical investigation documents to be maintained in sponsor and investigation site files is given in Annex E of ISO 14155:2020 standard.

24 FUNDING AND INSURANCE

24.1 Funding sources

The study was funded by a grant from the EIT (European institute for Innovation & Technology) Health and clinical centres, Synlab and ALCEDIAG.

24.2 Insurance

For the duration of the study, the Sponsor will take out an insurance policy covering the sponsor's own public liability, as well as the public liability for all the physicians involved in the study. The sponsor will also provide full compensation for any damages caused by the study to the participant enrolled and their beneficiaries, unless the sponsor can prove that the harm is not the fault of the sponsor or any collaborator. Compensation cannot be refused on the grounds of a third-party act or the voluntary withdrawal of the person who initially consented to participate in the study.

The sponsor has taken out insurance with CNA Insurance Company (Europe) S.A., which covers its own public liability and that of any collaborator (physician or research staff), in accordance with the applicable national and international laws and guidelines.

This study will be registered on the website http://clinicaltrials.gov/.

25 BIBLIOGRAPHY

1. Salvetat, N., et al., *A game changer for bipolar disorder diagnosis using RNA editing-based biomarkers.* Molecular Psychiatry, Submitted.

2. Lecrubier, Y., et al., *Mini International Neuropsychiatric Interview French Version 5.0.0.* 1998: p. INSERM-PARIS, FRANCE.
3. Montgomery, S.A. and M. Asberg, *A new depression scale designed to be sensitive to change.* Br J Psychiatry, 1979. **134**: p. 382-9.
4. Young, R.C., et al., *A rating scale for mania: reliability, validity and sensitivity.* Br J Psychiatry, 1978. **133**: p. 429-35.
5. Kim, S.H., et al., *Comparing the psychometric properties of the EQ-5D-3L and EQ-5D-5L in cancer patients in Korea.* Qual Life Res, 2012. **21**(6): p. 1065-73.
6. Hawass, N.E., *Comparing the sensitivities and specificities of two diagnostic procedures performed on the same group of patients.* Br J Radiol, 1997. **70**(832): p. 360-6.
7. DeLong, E.R., D.M. DeLong, and D.L. Clarke-Pearson, *Comparing the areas under two or more correlated receiver operating characteristic curves: a nonparametric approach.* Biometrics, 1988. **44**(3): p. 837-45.
8. Buderer, N.M., *Statistical methodology: I. Incorporating the prevalence of disease into the sample size calculation for sensitivity and specificity.* Acad Emerg Med, 1996. **3**(9): p. 895-900.
9. Merikangas, K.R., et al., *Prevalence and correlates of bipolar spectrum disorder in the world mental health survey initiative.* Arch Gen Psychiatry, 2011. **68**(3): p. 241-51.
10. Carvalho, A.F., et al., *Evidence-based umbrella review of 162 peripheral biomarkers for major mental disorders.* Transl Psychiatry, 2020. **10**(1): p. 152.
11. Carvalho, A.F., J. Firth, and E. Vieta, *Bipolar Disorder.* N Engl J Med, 2020. **383**(1): p. 58-66.
12. Fiedorowicz, J.G., J. He, and K.R. Merikangas, *The association between mood and anxiety disorders with vascular diseases and risk factors in a nationally representative sample.* J Psychosom Res, 2011. **70**(2): p. 145-54.
13. Pan, J.X., et al., *Diagnosis of major depressive disorder based on changes in multiple plasma neurotransmitters: a targeted metabolomics study.* Transl Psychiatry, 2018. **8**(1): p. 130.
14. Kessing, L.V., *Course and cognitive outcome in major affective disorder.* Dan Med J, 2015. **62**(11): p. B5160.
15. Giridharan, V.V., et al., *Postmortem evidence of brain inflammatory markers in bipolar disorder: a systematic review.* Mol Psychiatry, 2020. **25**(1): p. 94-113.
16. Maes, M. and A.F. Carvalho, *The Compensatory Immune-Regulatory Reflex System (CIRS) in Depression and Bipolar Disorder.* Mol Neurobiol, 2018. **55**(12): p. 8885-8903.
17. Mannion, N.M., et al., *The RNA-editing enzyme ADAR1 controls innate immune responses to RNA.* Cell Rep, 2014. **9**(4): p. 1482-94.
18. Lamers, M.M., B.G. van den Hoogen, and B.L. Haagmans, *ADAR1: "Editor-in-Chief" of Cytoplasmic Innate Immunity.* Front Immunol, 2019. **10**: p. 1763.
19. Rosenthal, J.J. and P.H. Seeburg, *A-to-I RNA editing: effects on proteins key to neural excitability.* Neuron, 2012. **74**(3): p. 432-9.
20. Berg, K.A., et al., *RNA-editing of the 5-HT(2C) receptor alters agonist-receptor-effector coupling specificity.* Br J Pharmacol, 2001. **134**(2): p. 386-92.
21. Yang, W., et al., *Altered RNA editing of serotonin 5-HT2C receptor induced by interferon: implications for depression associated with cytokine therapy.* Brain Res Mol Brain Res, 2004. **124**(1): p. 70-8.
22. Weissmann, D., et al., *Region-specific alterations of A-to-I RNA editing of serotonin 2c receptor in the cortex of suicides with major depression.* Transl Psychiatry, 2016. **6**(8): p. e878.
23. Chimienti, F., et al., *Brain region-specific alterations of RNA editing in PDE8A mRNA in suicide decedents.* Transl Psychiatry, 2019. **9**(1): p. 91.
24. Bender, A.T. and J.A. Beavo, *Cyclic nucleotide phosphodiesterases: molecular regulation to clinical use.* Pharmacol Rev, 2006. **58**(3): p. 488-520.

25. Salvetat, N., et al., *RNA editing blood biomarkers for predicting mood alterations in HCV patients.* J Neurovirol, 2019. **25**(6): p. 825-836.
26. Salvetat, N., et al., *Phosphodiesterase 8A to discriminate in blood samples patients with depression and suicide attempters from healthy controls based on A-to-I RNA editing modifications.* Transl Psychiatry, 2021. **11**(1): p. 255.
27. Wang, P., et al., *Seven novel mutations of ADAR in multi-ethnic pedigrees with dyschromatosis symmetrica hereditaria in China.* Mol Genet Genomic Med, 2019. **7**(10): p. e00905.
28. Jones, S.R., S. Carley, and M. Harrison, *An introduction to power and sample size estimation.* Emerg Med J, 2003. **20**(5): p. 453-8.

26 LIST OF ADDENDA

26.1 List of investigators

Address of the study location	Title	First name Surname	Telephone/E-mail/Fax
Hospital Clinic de Barcelona<br>Villarroel, 170 ; 08036 Barcelona<br>SPAIN	Pr	Eduard VIETA	+34-93-227-5477/<br>evieta@clinic.cat/<br>+34-93-227-9228
Capital Region of Denmark	Pr	Lars KESSING	+45 38 64 70 81<br>lars.vedel.kessing@regionh.dk
GHU Psychiatrie & Neurosciences	Pr	Chantal HENRY	+33 1 45 65 86 52<br>ch.henry@ghu-paris.fr
Parc Sanitari Sant Joan de Déu	Pr	Josep Maria HARO	+34 93 600 70 81<br>josepmaria.haro@sjd.es

26.2 Blood sampling and conservation

EDIT-B is made from a whole blood sample taken in a 2.5mL PAXgene® Blood RNA tube (Becton Dickinson). During this medical research, two tubes per patient are collected. Collection of blood samples can be performed at any time of the day. Each tube should be imperatively filled to the maximum. Labels and markers that are resistant to -80°C should be used.

Detailed steps for blood sample collection and their storage:
1. Label the PAXgene® tube with patient code.
2. Collect blood: If PAXgene® tube is the first tube to be drawn, use a discard tube firstly. Then fill the tube with 2.5mL of blood.
3. Ensure that the blood has stopped flowing into the tube.
4. Immediately after blood collection, gently invert tubes between eight and ten times.
5. Storage of the tubes upright in a wire rack for two hours at room temperature. Do not use expanded polystyrene foam.
6. Transfer the tubes upright and freeze them at -20°C during minimum three days until shipment.
7. Long-term storage at -80°C.

26.3 EDIT-B process

RNA extraction and qualification from whole blood
Samples are retrieved in PAXgene® blood RNA tubes, distributed randomly in different sets of extractions and isolated using MagNA Pure 96 instrument (Roche) (LifeScience), according to the manufacturer's protocol. Total RNA concentrations and quality levels are determined with Qubit Fluorometer (Invitrogen) and LabChip (Perkin-Elmer, HT RNA Reagent Kit) instruments, respectively. Only samples with RNA integrity values > 7 are taken into consideration for further analysis.

Targeted Next Generation Sequencing
Regions of interest for each gene are amplified with validated primers using Q5 Hot Start High Fidelity enzyme (New England Biolabs) according to manufacturer guidelines, on a Peqstar 96x thermocycler. PCR products are purified with magnetic beads (High Prep PCR MAGbio system, Mokascience), amplicon purity is determined with Nucleic Acid Analyzer (LabChipGx, Perkin Elmer) and then quantified (Qubit system, ThermoFisher Scientific). After indexing samples (Q5 Hot Start High fidelity PCR enzyme, Nextera XT index kit of Illumina), the library is pooled, purified (Magbio PCR cleanup system), denatured (0.1M NaOH), spiked with PhiX Control V3 (Illumina), loaded onto a sequencing cartridge (Illumina MiSeq Reagent Kit V3 or Illumina NextSeq 500/550 Mid-Output) according to Illumina's guidelines, and finally sequenced (1 x 150 bp read length) at standard concentrations using ultra-deep sequencing. Approximately 1 million reads per sample are acquired.

Bioinformatics analysis of targeted sequencing data
In this medical research, sequencing data will be analysed by Alcediag through a full secure Alcediag server using the three different signatures. For the intended use of the device, the sequencing data are uploaded by customer to the Alcediag server with EDITB_WEB and analysed by Alcediag with EDITB_AL, which gives a score, helping to differentiate if the patient is affected with bipolar disorder or unipolar depression. At the end, an analysis report with the diagnostic result of the test is delivered and downloaded by the client using EDITB_WEB. EDITB_AL and EDITB_WEB will be hosted on two different HDS (Health Data Hosting) certified servers. EDITB_WEB can securely connect and communicate to EDITB_AL using a REST API. REST APIs enable you to develop any kind of web application allowing to requesting systems to access and manipulate the textual representations of resources by using a uniform and predefined set of CRUD (Create, Retrieve, Update, Delete) operations.

20240515_EDIT-B_CPSP_v3_FRANCE_3-final

Final Audit Report 2024-05-29

Created:	2024-05-29
By:	Diana Vetter (diana.vetter@alcediag-alcen.com)
Status:	Signed
Transaction ID:	CBJCHBCAABAAo-4Yb8W3pJ3L3tPixgtFl1hykpVICu8w

"20240515_EDIT-B_CPSP_v3_FRANCE_3-final" History

- Document created by Diana Vetter (diana.vetter@alcediag-alcen.com)
  2024-05-29 - 10:05:55 AM GMT

- Document emailed to Eduard Vieta (evieta@clinic.cat) for signature
  2024-05-29 - 10:06:02 AM GMT

- Document emailed to Dinah WEISSMANN (dweissmann@alcediag-alcen.com) for signature
  2024-05-29 - 10:06:03 AM GMT

- Email viewed by Eduard Vieta (evieta@clinic.cat)
  2024-05-29 - 10:23:51 AM GMT

- Document e-signed by Eduard Vieta (evieta@clinic.cat)
  Signature Date: 2024-05-29 - 10:24:27 AM GMT - Time Source: server

- Email viewed by Dinah WEISSMANN (dweissmann@alcediag-alcen.com)
  2024-05-29 - 12:11:03 PM GMT

- Document e-signed by Dinah WEISSMANN (dweissmann@alcediag-alcen.com)
  Signature Date: 2024-05-29 - 12:11:20 PM GMT - Time Source: server

- Agreement completed.
  2024-05-29 - 12:11:20 PM GMT

Adobe Acrobat Sign
